# DeepMetabio-mCRC Screener: A Multi-Omics Deep Learning Framework for Early Risk Prediction and Biomarker Discovery in Colorectal Liver Metastasis

**DOI:** 10.34133/csbj.0074

**Published:** 2026-05-25

**Authors:** Hongyu Zhang, Ke Wang, Runqiu Guo, Xiaochuan Wu, Qingquan Chen, Qiaojun He, Bo Yang, Yanyan Zhuang, Wanling Yang, Hong Zhu

**Affiliations:** ^1^ Zhejiang Province Key Laboratory of Anti-Cancer Drug Research, College of Pharmaceutical Science, Innovation Institute for Artificial Intelligence in Medicine of Zhejiang University, Zhejiang University, Hangzhou 310058, China.; ^2^ Guangdong Provincial Key Laboratory of Malignant Tumor Epigenetics and Gene Regulation, Sun Yat-sen Memorial Hospital, Sun Yat-sen University, Guangzhou 510120, China.; ^3^ State Key Laboratory of Advanced Drug Delivery and Release Systems, Institute of Pharmaceutics, College of Pharmaceutical Sciences, Zhejiang University, Hangzhou 310058, China.; ^4^ School of Medicine, Hangzhou City University, Hangzhou, 310015, China.; ^5^ Department of Paediatrics and Adolescent Medicine, The University of Hong Kong, Hong Kong, China.

## Abstract

Colorectal liver metastasis (CRLM) remains the primary cause of mortality in patients with colorectal cancer (CRC), yet effective predictive tools and reliable biomarkers are still lacking. DeepMetabio-mCRC Screener, an integrated multi-omics framework combining large-scale transcriptomic profiles with serum metabolomics, was developed to address this gap. In a cohort of 1,077 CRC samples, 620 metabolism-related genes were used to train a convolutional neural network, yielding an area under the receiver operating characteristic curve of 0.92 in the validation cohort and 0.97 in the independent testing cohort, outperforming the performance of the 10 established machine learning models. Model-derived transcriptomic risk scores revealed 22 core metabolic features associated with metastatic progression and CRLM occurrence, particularly retinol and tryptophan metabolism. Cross-omics integration revealed aminocarboxymuconate-semialdehyde decarboxylase (ACMSD) as a promising biomarker associated with impaired nicotinamide adenine dinucleotide biosynthesis. Clinical validation in 100 CRC patients confirmed elevated ACMSD levels in patients with CRLM, which correlated with advanced stage, recurrence risk, an immune-inflamed tumor microenvironment, and heightened sensitivity to epidermal growth factor receptor/vascular endothelial growth factor receptor-targeted therapies. In vitro, ACMSD knockdown was associated not only with suppressed CRC cell migration caused by inhibition of the transforming growth factor-β/epithelial-to-mesenchymal transition pathway but also with decreased proinflammatory and immune-responsive pathways and reduced immune cell infiltration. These findings collectively validate the DeepMetabio-mCRC Screener as a substantial early risk prediction tool and underscore ACMSD, identified through this framework, as a multifunctional biomarker for diagnosis, prognosis, molecular characterization, and therapeutic decision-making in patients with CRLM.

## Highlights

• DeepMetabio-mCRC Screener, a 1-dimensional convolutional neural network (CNN) integrating multicohort transcriptomic profiles, was developed for robust early detection of metastatic colorectal cancer (mCRC).
• Model-derived transcriptomic risk scores (TRS) identified 22 core metabolic features enriched in retinol and tryptophan metabolism, highlighting key pathways in colorectal liver metastasis (CRLM).
• Cross-omics integration identified ACMSD as a diagnostic and prognostic biomarker that mechanistically impaired NAD^+^ biosynthesis and an immune-inflamed tumor microenvironment (TME).
• Clinical and in vitro validation demonstrated that ACMSD predicts CRLM risk, postoperative recurrence, metastatic mechanisms, and therapeutic responsiveness to EGFR/VEGFR-targeted agents, supporting its application in personalized treatment strategies.

## Graphical Abstract

**Figure:**
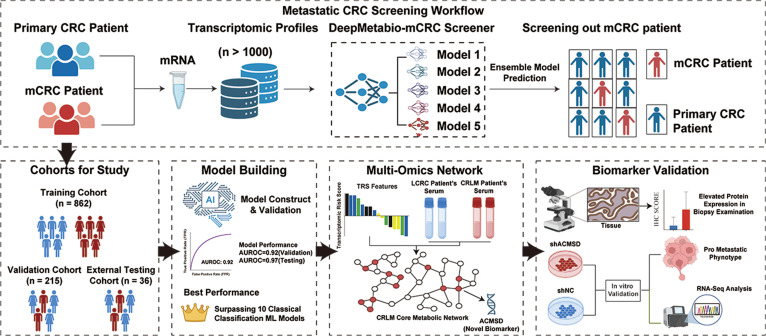

## Introduction

Colorectal cancer (CRC) is the third most commonly diagnosed malignancy worldwide, with approximately 2 million new cases and nearly 1 million deaths reported annually [1]. Metastatic CRC (mCRC) is associated with poor prognosis, with a survival rate less than 15% [2]. Within 5 years of surgery, nearly half of CRC patients develop metastatic disease [3]; 25% to 30% present with synchronous metastases at diagnosis, and 15% to 20% subsequently develop metachronous lesions [4]. Liver metastases occur in 50% to 60% of mCRC cases and represent the leading cause of mortality [5]. Without treatment, the median survival time (MST) for patients with liver metastases is typically less than 12 months, while aggressive interventions extend the MST to only 13 to 18 months [6].

Therapeutic advances, including targeted agents such as epidermal growth factor receptor (EGFR) and vascular endothelial growth factor receptor (VEGFR) inhibitors in combination with chemotherapy and surgical resection, have improved the survival of patients with mCRC [7]. However, untreated liver metastases continue to yield an MST of 6.9 months and a 5-year survival rate of less than 5% [8]. In contrast, complete resection with no evidence of disease extends the MST to 35 months and increases 5-year survival rates to 30% to 57% [2]. These findings underscore the importance of early detection of colorectal liver metastases (CRLMs), as timely identification can facilitate the conversion of unresectable lesions to a resectable or no-evidence-of-disease status, thereby improving long-term outcomes [9].

Despite therapeutic progress, early screening for mCRC remains limited. Current CRLM diagnostic approaches rely primarily on imaging modalities, such as colonoscopy, liver ultrasound, contrast-enhanced abdominal computed tomography, and serum tumor markers such as serum carcinoembryonic antigen and carbohydrate antigen 19-9 [10,11]. These methods are effective for detecting established metastases but offer limited predictive value for assessing metastatic risk or elucidating biological mechanisms. Although genomic alterations such as mutations in APC, TP53, KRAS, and BRAF provide prognostic and therapeutic information in established mCRC, biomarkers that predict CRLM development are rare [8,12]. Furthermore, genomic-only strategies insufficiently capture the metabolic reprogramming and tumor–microenvironment interactions that are fundamental to metastatic progression.

To address these challenges, we developed an integrated multi-omics framework combining large-scale transcriptomic profiling, deep learning-based modeling, and serum metabolomics for the early detection of mCRC. Through this approach, aminocarboxymuconate-semialdehyde decarboxylase (ACMSD) was identified as a promising metabolic biomarker with demonstrable discriminatory power. By serving as a regulator of nicotinamide adenine dinucleotide (NAD^+^) metabolic reprogramming and being associated with transforming growth factor-β (TGF-β)-mediated metastasis, immune modulation, and drug sensitivity, ACMSD has demonstrated substantial potential as a versatile biomarker for risk prediction, risk stratification, and personalized therapeutic decision-making in patients with CRLM.

## Materials and Methods

### Preprocessing of CRC gene expression datasets

CRC gene expression datasets, comprising mRNA profiles with associated clinical annotations, were primarily obtained from the Gene Expression Omnibus (GEO) database (https://www.ncbi.nlm.nih.gov/geo/). RNA sequencing (RNA-seq) data for The Cancer Genome Atlas (TCGA) and Sidra-LUMC AC-ICAM cohort (SILU), normalized per kilobase of transcript per million mapped reads (FPKM), together with corresponding clinical information, were retrieved from the cBioPortal platform (https://www.cbioportal.org/).

For the GEO datasets, GSE131418 (749 samples, GPL15048 platform) was normalized using iterative rank-order normalization with tools available at http://gene.moffitt.org/libaffy/. Datasets on the GPL570 platform (GSE81986, GSE41568, GSE71222, GSE18105, GSE21510, GSE27854, GSE72970, and GSE17536) were processed using the robust multiarray average method implemented in the R package Affy. Additionally, GSE204805 (119 samples, GPL18573 platform) and GSE50760 (36 samples, GPL11154 platform) were subjected to FPKM normalization.

The input features were predefined using an independent discovery cohort (GSE131418) to ensure that feature selection remained blind to the development and testing cohorts.

Datasets were assigned to either internal cohorts (COAD-SILU, GSE18105, GSE21510, GSE27854, GSE41568, GSE71222, GSE81986, and GSE204805) or a strictly independent external test cohort (GSE50760). Unified exclusion criteria were applied: removal of samples with prior systemic therapy, exclusion of normal/adjacent and nonhuman tissues, and deduplication across all cohorts. Importantly, to strictly prevent data leakage and ensure rigorous evaluation, we enforced patient-level isolation between the model development and testing phases. We confirm that the patients comprising the external testing cohort (GSE50760) were entirely mutually exclusive from those in the training and internal validation cohorts, ensuring zero patient-level overlap. Detailed dataset characteristics are provided in Table S2.

### Construction of the DeepMetabio-mCRC Screener model

#### Sample integration, preprocessing, and batch effect correction

Multiple publicly available CRC transcriptomic datasets, including microarray profiles (GPL570 platform) and RNA-seq data (Illumina NGS), were integrated. For each dataset, gene expression matrices and associated clinical metadata were retrieved, harmonized, and merged by retaining only genes that were common to all cohorts. The originating platform was annotated as the batch variable, and disease status (primary versus metastatic) was defined as the biological grouping factor. To mitigate nonbiological variation, we adjusted cross-platform batch effects using the ComBat function from the sva R package, with disease status included as a covariate. Importantly, GSE50760 was withheld from all data integration and batch-effect correction (ComBat) workflows to ensure model independence. Correction efficacy was evaluated using silhouette scores (Cluster R package), with lower values indicating improved interplatform mixing.

#### Model development, training, and evaluation

For model input, 620 metabolism-related genes were selected from the batch-corrected dataset. Disease status was encoded as binary labels (0, primary; 1, metastasis). The dataset was stratified and randomly divided into training and independent test sets at an 80:20 ratio. *z*-score standardization parameters were estimated from the training data within each fold and subsequently applied to the validation and test sets to prevent information leakage.

The DeepMetabio-mCRC Screener was implemented as a 1-dimensional convolutional neural network (1D-CNN) using TensorFlow (v2.10) and Keras frameworks. The network architecture consisted of sequential modules. The first convolution–pooling block contained a Conv1D layer with 32 filters, a kernel size of 5, and rectified linear unit (ReLU) activation [13], where the activation function was defined as follows:

$$f\left(x\right)=\max\left(0,\;x\right)$$ (1)

This was followed by a MaxPooling1D layer with a pool size of 2 and a dropout layer (rate 0.3) to mitigate overfitting. The second convolution–pooling block included a Conv1D layer with 64 filters, a kernel size of 5, and ReLU activation, followed by a MaxPooling1D layer (pool size 2) and a dropout layer (rate 0.4). The fully connected module comprised a flattened layer, a dense layer with 64 neurons and ReLU activation, and a dropout layer (rate 0.5). The output layer consisted of a single dense neuron with sigmoid activation [14], defined as follows:

$$f\left(x\right)=1/\left(1+e\;\hat{\;}\;\left(-x\right)\right)$$ (2)

which yielded a probability score (0 to 1) for metastatic classification.

The model was compiled with the Adam optimizer, an initial learning rate of 0.001, and the binary cross-entropy loss function [15], expressed as follows:

$$\text{Loss}=-\left(y^{*}\;\log\left(p\right)+{\left(1-y\right)}^{*}\;\log\left(1-p\right)\right)$$ (3)

where *y* denotes the true label and *p* represents the predicted probability of the positive class.

Model training and validation followed a 5-fold stratified cross-validation strategy to maintain balanced class distributions. Two callback functions were employed during training: Early Stopping, which monitored validation loss (val_loss) with a patience of 10 epochs, and Reduce LR On Plateau, which reduced the learning rate by a factor of 0.5 if the validation loss failed to improve after 5 consecutive epochs.

Upon completion of cross-validation, the 5-fold-specific models were retained. For inference on the independent test set, the predicted probabilities from the 5 models were averaged to generate the ensemble predictions.

Model performance was assessed on both the validation folds and the independent test set using accuracy, precision, sensitivity (recall), and the area under the receiver operating characteristic (ROC) curve (AUC) as the primary performance metrics. All procedures were conducted in Python (v3.9.13) with fixed random seeds (SEED = 42) to ensure reproducibility.

### Benchmarking against conventional machine learning models

To contextualize the predictive performance of the DeepMetabio-mCRC Screener, benchmarking was performed against 10 widely used machine learning classifiers, representing major algorithmic families that are commonly applied in biomedical research. These include (a) linear models—logistic regression (LR, with sigmoid transformation); (b) kernel-based methods—support vector machine (radial basis function kernel); (c) instance-based learning—K-nearest neighbors; (d) probabilistic classifiers—Gaussian naïve Bayes (assuming feature independence with Gaussian distribution per class); and (e) ensemble methods—random forest, decision tree, gradient boosting, adaptive boosting (AdaBoost), light gradient boosting machine (LightGBM, employing gradient-based 1-side sampling and exclusive feature bundling), and extreme gradient boosting (XGBoost, with L1/L2 regularization and parallelized tree building). Model selection ensured a broad representation of the statistical and algorithmic paradigms that are extensively used in bioinformatics and clinical prediction.

All the comparative models were trained and evaluated using the same datasets and features as the DeepMetabio-mCRC Screener. Performance was assessed using the area under the ROC curve (AUC) as the primary metric, along with accuracy, sensitivity (recall), specificity, and precision. The implementations were conducted in Python (v3.9) using scikit-learn (v1.1), lightgbm (v3.3), and xgboost (v1.6), with fixed random seeds to ensure reproducibility.

### Transcriptomic risk score calculation and interpretation

The transcriptomic risk score (TRS) was computed using an ensemble of 5 convolutional neural network (CNN) models derived from 5-fold stratified cross-validation. For each sample, the expression profile of 620 input features was extracted as the input vector x, standardized using the fold-specific scaler, and processed by the corresponding model to yield a probability score Pi(metastasis∣x)∈[0,1], which represented the likelihood of metastatic classification.

The TRS was defined as the arithmetic mean of the 5 model outputs:

$$\mathrm{TRS}=\frac{1}{5}\sum_{i=1}^{5}P_{i}\left(\text{metastasic}\left|x\right.\right)$$ (4)

where *P_i_* is the predicted probability from the *i*^th^ model. Higher TRS values indicate greater predicted metastatic risk. In practice, a default threshold of TRS ≥ 0.5 can be applied to stratify samples into high-risk metastatic (TRS ≥ threshold) or low-risk primary (TRS < threshold) groups. TRS values for all the samples across the training and validation cohorts were compiled with corresponding true and predicted labels for downstream statistical analysis, biomarker correlation, and visualization.

While a default cutoff of TRS ≥ 0.5 was utilized for baseline performance evaluation during model development, the optimal threshold for independent external validation was determined using the data-driven Youden index to maximize predictive accuracy and account for potential distribution shifts across different platforms.

### Differential gene expression and functional analysis

Differential expression analysis was conducted using the R package limma (version 3.58.1). Differentially expressed genes (DEGs) were identified with thresholds of |log_2_FC| ≥ 1 and a *P* value < 0.05. Functional enrichment analyses, including Gene Ontology and Kyoto Encyclopedia of Genes and Genomes (KEGG) pathway enrichment analyses, were performed using the R package clusterProfiler (version 4.10.1). Gene set enrichment analysis (GSEA) and gene set variation analysis (GSVA) were conducted using clusterProfiler and the R package GSVA (version 1.50.5), respectively.

### Immune TME analysis and immunotherapy prediction

The tumor microenvironment (TME) was analyzed using the R package ESTIMATE (version 1.0.13) to calculate Estimation of STromal and Immune cells in MAlignant Tumor tissues using Expression data (ESTIMATE), immune, stromal, and tumor purity scores, reflecting immune infiltration and tumor malignancy. To further deconvolute immune and stromal components within the TME, the R package IOBR (version 0.99.8) was employed.

The correlation between CRLM biomarkers and immune cell infiltration was assessed using the single-sample GSEA (ssGSEA) function in the GSVA package (version 1.50.5). Significant correlations (*P* value < 0.05) were visualized as heatmaps generated with the pheatmap package (version 1.0.12).

### Prognostic analysis

Survival analysis was performed using the R package survival (version 3.5.8). Kaplan–Meier survival curves were generated and visualized with the R package survminer (version 0.4.9). The optimal cutoff points for continuous variables were determined using the surv_cutpoint function in survminer to stratify patients into distinct risk groups.

### Patient sample collection

Postoperative tissue samples were obtained from pathological specimens at Sun Yat-sen Memorial Hospital (Department of Pathology) between 2019 and 2024. The specimens were stored at 4 °C and reevaluated by a pathologist prior to immunohistochemical (IHC) staining. Pathology reports served as the primary reference for sample classification.

Peripheral blood samples were collected from CRC patients using red-capped tubes without anticoagulants (Table S27). A total of 4 ml of blood was allowed to coagulate at 37 °C or room temperature for 2 h, followed by centrifugation at 3,000 rpm for 15 min at 4 °C. The resulting serum was aliquoted into 1.5-ml tubes, stored at −80 °C, and transported on dry ice for metabolomic analysis.

This study was approved by the Medical Ethics Committee of Sun Yat-sen Memorial Hospital (SYSKY-2024-704-01), and informed consent was obtained from all participants.

### Immunohistochemistry

Paraffin-embedded tissue sections were deparaffinized in xylene and rehydrated through a graded series of ethanol. Antigen retrieval was performed in 1× tris-EDTA buffer (pH 9.0) via microwave heating, followed by cooling and washing with phosphate-buffered saline (pH 7.4). Endogenous peroxidase activity was blocked with 3% hydrogen peroxide for 25 min at room temperature. The sections were then blocked with 3% bovine serum albumin for 30 min before being incubated overnight at 4 °C with the anti-ACMSD primary antibody (Sigma, HPA011179, 1:100).

After being washed, the slides were incubated with a horseradish peroxidase-conjugated secondary antibody for 50 min and developed with 3,3′-diaminobenzidine under microscopic observation. Nuclear counterstaining was performed with hematoxylin, followed by differentiation, bluing, dehydration, and mounting. Stained sections were imaged for subsequent analysis.

ACMSD expression was quantified using ImageJ software with the IHC Profiler plugin. At least 6 representative fields per section were analyzed. The IHC score was calculated as the product of the cytoplasmic staining intensity and the percentage of positively stained cells. The percentages of positive cells were graded as follows: 0 (<5%), 1 (6% to 25%), 2 (26% to 50%), 3 (51% to 75%), and 4 (>75%). Cytoplasmic staining intensity was categorized into 4 levels: 0 (negative), 1 (weak), 2 (moderate), and 3 (strong) [16,17].

### Sample preparation and liquid chromatography-tandem mass spectrometry analysis

Samples stored at −80 °C were thawed on ice, vortexed briefly, and mixed with 300 μl of extraction solution (acetonitrile:methanol = 1:4, v/v) containing internal standards. After vortexing for 3 min and centrifugation (12,000 rpm, 10 min, 4 °C), 200 μl of the supernatant was collected, cooled at −20 °C for 30 min, and centrifuged again, and 180 μl of the resulting supernatant was subjected to liquid chromatography-mass spectrometry (LC-MS) analysis.

LC-tandem MS was performed on a QTRAP system equipped with a Waters HSS T3 C18 column (2.1 mm × 100 mm, 1.8 μm). Gradient elution was carried out at 0.4 ml/min and 40 °C using 0.1% formic acid in water (mobile phase A) and acetonitrile (mobile phase B). The gradient increased from 5% to 99% B over 6 min, followed by reequilibration.

The QTRAP system operated in both positive and negative ion modes with an ESI Turbo Ion-Spray interface. The source conditions included 500 °C, 5,500 V (positive mode), −4,500 V (negative mode), and high-collision gas. Instrument tuning and mass calibration were performed using 10 and 100 μmol/l polypropylene glycol solutions in triple quadrupole and linear ion trap modes. Multiple reaction monitoring transitions were optimized for metabolite detection.

### Differential metabolite analysis

Differential metabolites (DEMs) between 2 groups were identified using variable importance in projection (VIP) values greater than 1 (VIP > 1) and the Student *t* test, with significance defined as a *P* value < 0.05. VIP values were extracted from orthogonal partial least squares discriminant analysis (OPLS-DA), which also generated score and permutation plots using the R package MetaboAnalystR. OPLS-DA permutation testing is explicitly stated that the metabolomics data underwent log2​ transformation and mean centering prior to OPLS-DA modeling, and a 200-iteration permutation test was performed to evaluate the model and prevent overfitting.

### Cell culture

The CRC cell lines SW480 and NCI-H716 were obtained from the Cell Bank of the Chinese Academy of Sciences (Shanghai, China). Both cell lines were maintained in a controlled CO₂-independent environment without CO₂ at 37 °C. SW480 cells were cultured in Leibovitz’s L-15 medium (Gibco, Waltham, MA, USA), while NCI-H716 cells were cultured in RPMI 1640 medium (Gibco, Waltham, MA, USA). Both media were supplemented with 10% fetal bovine serum (Gibco).

### Migration evaluation

Cell migratory capacity was evaluated using a Transwell migration assay (24-well format; pore size: 8 μm; Sigma, Germany) without Matrigel precoating. Briefly, SW480 cells subjected to the indicated pretreatments were resuspended in serum-free L-15 medium at a density of 2 × 10^5^ cells/200 μl and seeded into the upper chamber. The lower chamber was filled with 700 μl of complete medium supplemented with 10% fetal bovine serum as a chemoattractant. Following a 72-h incubation at 37 °C, nonmigrated cells on the upper surface of the membrane were gently removed with a cotton swab. Cells that had migrated to the lower surface were fixed with 4% paraformaldehyde for 2 h and stained with 0.1% crystal violet for 30 min. Five random microscopic fields per membrane were imaged using an inverted microscope (Olympus, Japan), and migrated cells were quantified. Each experiment was performed in triplicate.

### shRNA knockdown and transfection assays

Small hairpin RNA (shRNA) sequences targeting the gene of interest were purchased from GuanNan Biotech (Hangzhou, China). Lentiviral particles were generated by cotransfecting 293T packaging cells with the shRNA expression vector and helper plasmids [18] using polyethylenimine (MCE, HY-K2014) following the manufacturer’s protocol. For transduction, the cells were incubated with viral supernatants for 48 h, after which the medium was replaced with fresh growth medium. At 72 h postinfection, puromycin (5 μg/ml) was applied for 24 h to select stably transduced cells.

### RNA-seq and data analysis

Total RNA from NCI-H716 cells (shACMSD and shNC groups) was extracted, and cDNA libraries were constructed using the SMARTer cDNA synthesis method, followed by double-stranded DNA fragmentation. Paired-end sequencing (2 × 150 bp) was performed on an Illumina NovaSeq 6000 platform (LC Sciences, USA). Raw reads were quality-filtered using Cutadapt (v1.9) to remove adapters, polyA/G sequences, unknown nucleotides (>5%), and low-quality bases (>20% *Q*-value ≤ 20) [19]. Clean reads were then aligned to the human reference genome using HISAT2 (v2.2.1) [20]. Gene abundance was quantified by StringTie (v2.1.6) to calculate FPKM values [21].

### Bioinformatics tools and statistical analysis

All the statistical analyses and data visualizations were performed in the R environment (version 4.3.2). Comparisons between 2 independent groups were conducted using the Student *t* test or the Wilcoxon rank-sum test when assumptions of normality or homogeneity of variance were not met. Comparisons among multiple groups were performed using 1-way analysis of variance (ANOVA) for normally distributed data with equal variances or the Kruskal–Wallis test otherwise. Associations between continuous variables were assessed using Pearson or Spearman correlation coefficients, as appropriate. Statistical significance was set at *P < 0.05, **P < 0.01, and ***P < 0.001.

## Results

### Research strategy and identification of input features

**Fig. 1.**
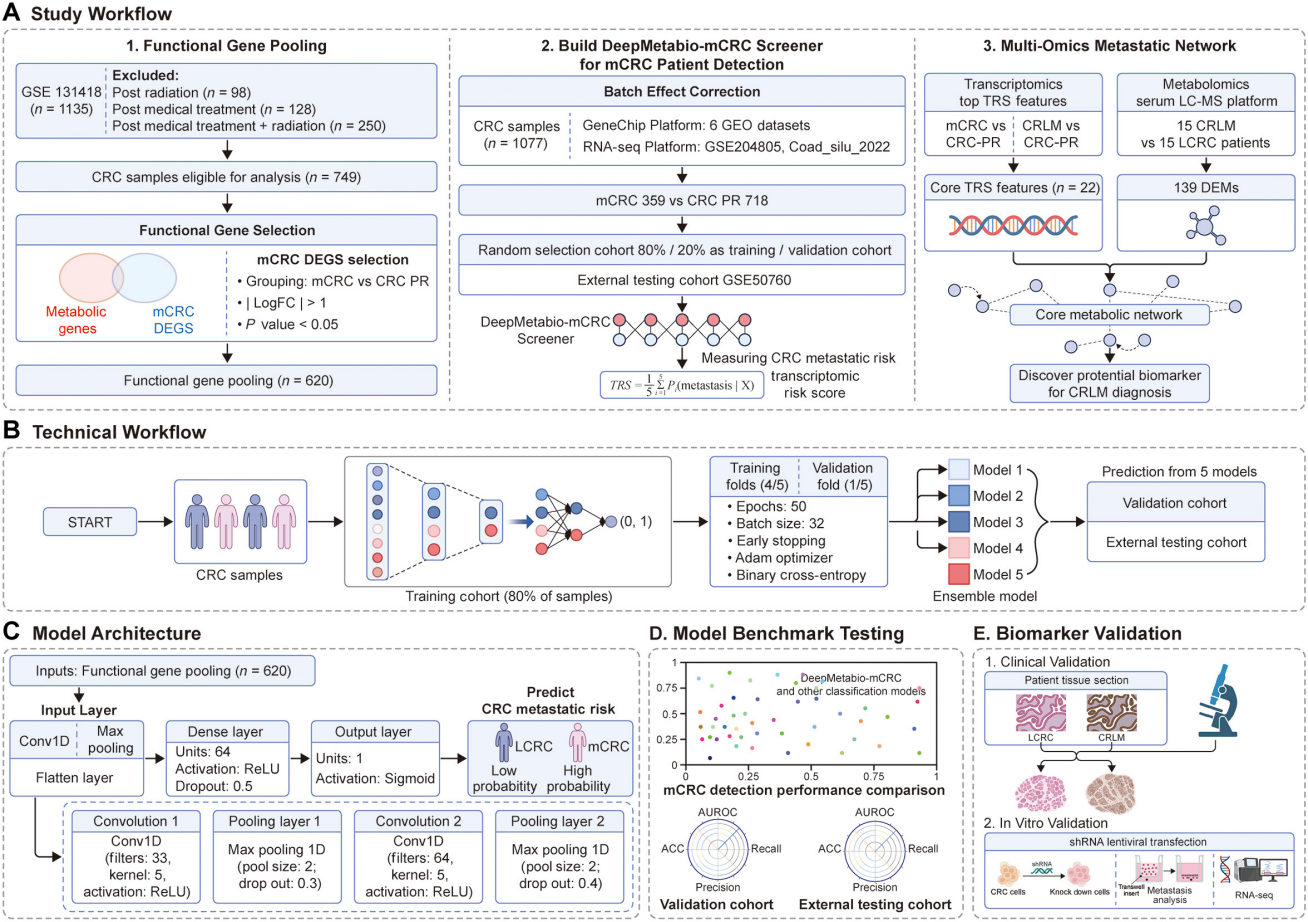
Overview of the study workflow for early metastatic colorectal cancer detection and biomarker validation. (A) Comprehensive study workflow. The study comprised 3 stages. 1. Functional Gene Pooling: Differentially expressed genes (DEGs) between metastatic colorectal cancer (mCRC) and primary colorectal cancer (CRC-PR) samples (GSE131418, *n* = 749 after exclusions) were identified and intersected with metabolic genes to form a functional gene pool (*n* = 620). 2. DeepMetabio-mCRC Screener Development: Multiplatform transcriptomic datasets (*n* = 1,077) were integrated, batch-corrected, and used with the functional gene pool to build, train, validate, and externally test the DeepMetabio-mCRC Screener for early mCRC detection. 3. Multi-Omics Metastatic Network: Model outputs and transcriptomic risk score (TRS) features (*n* = 22) were integrated with serum metabolomics (975 DEMs from 15 colorectal liver metastases [CRLMs] versus 15 localized CRCs [LCRCs]) to construct a cross-omics network for identifying CRLM early-screening biomarkers. (B) DeepMetabio-mCRC Screener Technical Workflow. 1,017 CRC samples were split into 80% training and 20% validation cohort. The training cohort used 5-fold stratified cross-validation (4 folds for training, 1 for validation). The model was trained with Adam optimizer (LR=0.001), binary cross-entropy, 50 epochs (batch size=32), and early stopping (patience=10). Performance was evaluated by area under the receiver operating characteristic curve (AUC), accuracy, precision, recall, and specificity. Five fold-specific models were ensembled for prediction. (C) DeepMetabio-mCRC Screener Model Architecture. The input layer used 620 standardized functional gene expressions. Two convolution-pooling blocks followed: the first with Conv1D (32 filters, kernel=5, rectified linear unit [ReLU]), MaxPooling1D (pool=2), and Dropout (0.3); the second with Conv1D (64 filters, kernel=5, ReLU), MaxPooling1D (pool=2), and Dropout (0.4). The flattened convolutional output passed to a Dense layer (64 neurons, ReLU, Dropout=0.5), leading to a single Sigmoid output neuron predicting metastatic risk (0 to 1). (D) Benchmark Testing. A scatter plot illustrates the model’s detection comparison. Bar graphs quantify the DeepMetabio-mCRC Screener’s performance (AUC, accuracy, precision, and recall) against other classifiers in validation and external testing cohorts for mCRC patient identification. (E) Biomarker Validation Workflow. This outlines the validation of CRLM early-screening biomarkers. 1. Clinical Validation: Assessment in patient tissue sections (CRLM versus LCRC) for clinical feasibility. 2. In Vitro Validation: Evaluation in CRC cell models via RNA sequencing (RNA-seq) and small hairpin RNA (shRNA)/lentivirus transfection to determine functional roles. Images were created with BioRender.

To predict mCRC occurrence and assess associated metabolites, extensive transcriptomic and metabolic gene analyses were performed on patients with CRC. In accordance with the study workflow (Fig. 1A), 749 mCRC and primary CRC (CRC-PR) samples were analyzed after patients who had received prior drug or radiotherapy treatment were excluded. Differential expression analysis revealed mCRC-specific genes using thresholds of log_2_FC > 1 or log_2_FC < −1 and a *P* value < 0.05 (Table S2). Intersection of these genes with a curated metabolic reprogramming gene set [22] yielded 620 functional gene pooling members (Table S3).

Integrated transcriptomic profiles from 8 datasets (GeneChip GPL570 with robust multiarray average normalization; RNA-seq Illumina NGS with log_2_ + 1 transformation), totaling 1,077 samples, were batch-corrected and partitioned into training (80%) and validation (20%) sets. Using 620 functional genes as inputs, the DeepMetabio-mCRC Screener was constructed, which demonstrated stable performance during internal validation and favorable accuracy for early mCRC detection in the external GSE50760 test set. Model-derived TRSs were used to identify the top 50 contributing features. Among these, 22 “core TRS features” overlapped significantly between the mCRC and CRLM cohorts and were enriched in specific metabolic pathways. Serum metabolomics (LC-MS) from 30 patients with CRC (15 patients with CRLM versus 15 patients with CRC-PR) revealed 139 differentially abundant metabolites. Integration of transcriptomic and metabolomic pathways enabled the construction of a cross-omics network, highlighting key blood metabolic pathways and candidate biomarkers for early CRLM screening.

Principal component analysis (PCA) clearly distinguished between the mCRC and CRC-PR groups (Fig. S1A). A total of 4,067 mCRC-associated DEGs were identified (Fig. S1B), of which 620 intersected with human metabolic genes and were linked to mCRC progression (Fig. S1C). Precorrection PCA revealed dataset-specific clustering with pronounced interbatch variation (Fig. S1D). Following ComBat correction, interbatch effects were markedly reduced as samples intermixed in PCA space (Fig. S1E). The mean silhouette score of all the samples (Table S4) decreased below 0.1 following correction (Fig. S1F), confirming a substantial reduction in batch effects (Table S5).

### Model construction, training, and validation

Building upon the curated panel of 620 metabolism-related features obtained from functional gene pooling and batch-corrected transcriptomic profiles, the DeepMetabio-mCRC Screener was developed as an end-to-end predictive framework for distinguishing mCRC from CRC-PR samples. The multistage workflow (Fig. 1B) included data preprocessing, stratified partitioning into development and test sets, and 5-fold cross-validation. The CNN architecture (Fig. 1C) extracted hierarchical transcriptomic features through sequential convolution–pooling layers, fully connected layers, and a sigmoid output. The training strategy employed supervised optimization with binary cross-entropy loss, and the Adam optimizer was over 50 epochs using a batch size of 32.

**Fig. 2.**
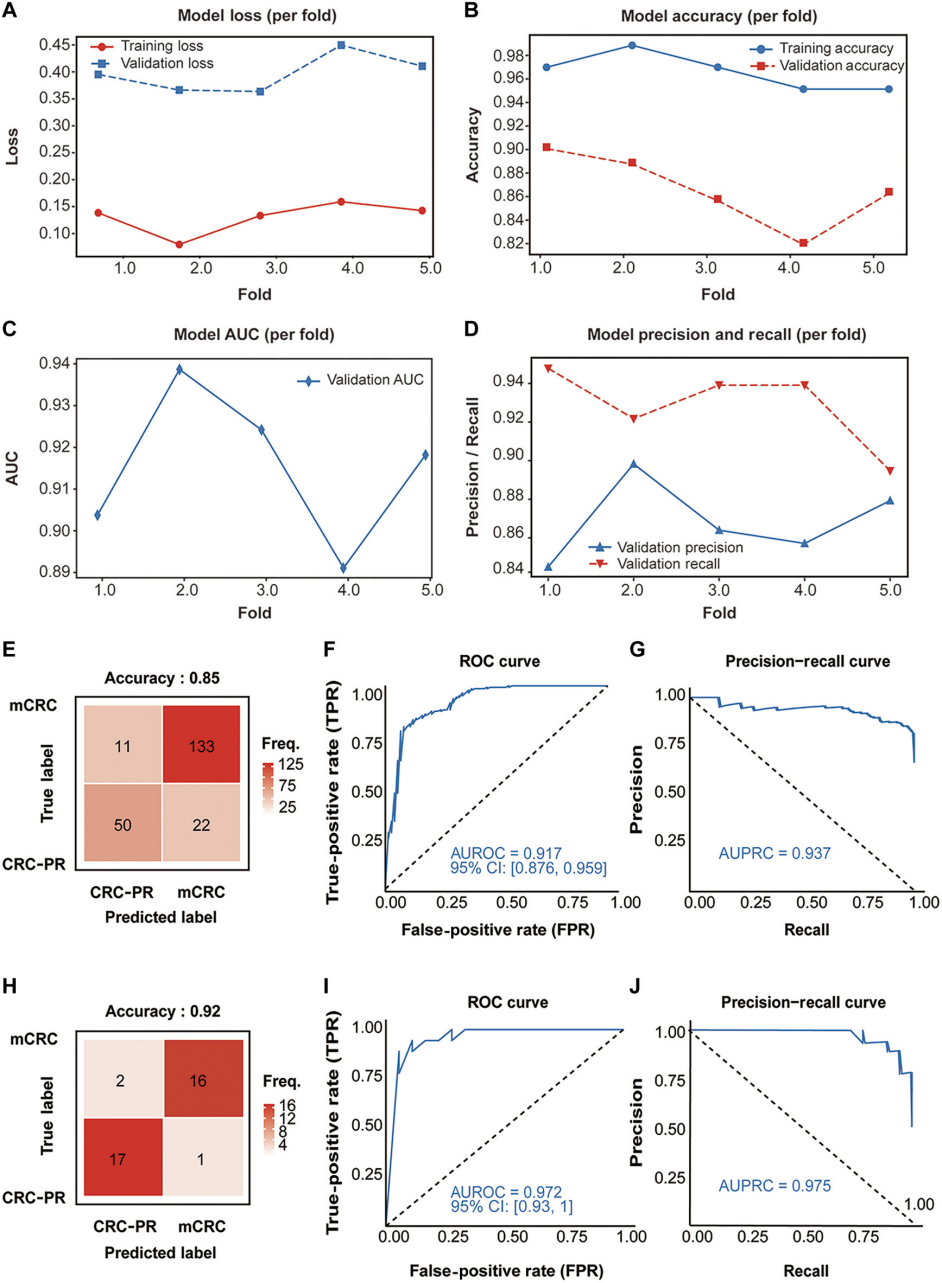
Training, internal validation, and external validation performance of the DeepMetabio-mCRC Screener. (A) Loss curves of each fold on the training and validation sets across epochs, used to evaluate training convergence and potential overfitting. (B) Accuracy curves of each fold on the training and validation sets. (C) Area under the receiver operating characteristic curve (AUC) for each fold on the validation set. (D) Precision and recall metrics for each fold on the validation set. (E) Confusion matrix for the internal validation set. (F and G) Receiver operating characteristic curve (F) and precision–recall curve (G) for the internal validation set, with the corresponding AUC values. (H) Confusion matrix for the external test cohort (CRLM). (I and J) Receiver operating characteristic curve (I) and precision–recall curve (J) for the external testing set, with the corresponding AUC values. CI, confidence interval.

During 5-fold cross-validation, the Screener showed stable convergence. Training and validation losses decreased steadily (Fig. 2A) and plateaued without divergence in accuracy (Fig. 2B), indicating no evidence of overfitting. Consistent ROC curves (Fig. 2C) and precision–recall curves (Fig. 2D) across folds demonstrated strong and stable discriminative capacity (Table S6).

Internal cross-validation yielded substantial classification performance: accuracy = 0.85 (Fig. 2E), AUC = 0.92 (Fig. 2F), precision = 0.86, and recall (sensitivity) = 0.92 (Fig. 2G), indicating reliable and highly sensitive mCRC identification (Table S7). External validation on the independent test set confirmed the comparable results: accuracy = 0.92 (Fig. 2H), AUC = 0.97 (Fig. 2I), precision = 0.94, and recall (sensitivity) = 0.88 (Fig. 2J). Despite minor fluctuations, the high discriminative ability highlighted the model stability and generalizability across independent samples (Table S8). To comprehensively evaluate the model’s robustness under extreme class imbalance and cross-platform heterogeneity, extended Leave-One-Dataset-Out (LODO) validation metrics, including balanced accuracy, PR-AUC, and Brier scores, were computed and detailed in Table S9.

Collectively, these results established the DeepMetabio-mCRC Screener as a reliable tool for accurately identifying patients with CRC at elevated metastatic risk by capturing underlying transcriptomic signatures.

### The DeepMetabio-mCRC Screener exhibits favorable and stable generalization

To evaluate classification performance and generalizability, the DeepMetabio-mCRC Screener was benchmarked against 10 classical machine learning classifiers: LightGBM, random forest, gradient boosting, XGBoost, AdaBoost, support vector machine, LR, K-nearest neighbors, decision tree, and Gaussian naïve Bayes. All the models were trained on the same datasets and evaluated on both the internal validation set and an independent external test set.

With respect to the internal validation set (Fig. S2A), most of the models, including LightGBM and random forest, achieved high accuracy, AUC, recall (sensitivity), and precision and performed comparably to the DeepMetabio-mCRC Screener. However, a marked decline in performance was observed for nearly all the classical models on the external test set (Fig. S2B); LightGBM exhibited the steepest decrease, while random forest also demonstrated substantial degradation. In contrast, the DeepMetabio-mCRC Screener maintained a performance that was nearly identical to that of the internal validation, outperforming all the comparators across the 4 evaluation metrics (Fig. S2C and Table S10).

These findings indicated that while conventional models lacked robustness on independent data, the DeepMetabio-mCRC Screener maintained stable, high-level performance across datasets, underscoring its strong generalizability and potential clinical utility.

### Establishment and prospective clinical validation of the TRS system

Building upon the DeepMetabio-mCRC Screener, a TRS system was developed to interpret model outputs and quantify feature contributions to CRC metastasis risk. TRS was calculated as the mean predicted probability of metastasis across 5 parallel submodels (see Materials and Methods), with higher values reflecting increased metastatic potential.

To evaluate the prospective predictive capacity and longitudinal risk stratification ability of the TRS, 3 independent cohorts with long-term follow-up data (GSE39582, GSE17536, and TCGA-COADREAD) were analyzed. To strictly assess early risk prediction, patients with established distant metastasis (Pathological M1/Stage IV) at diagnosis were systematically excluded, restricting the analysis exclusively to initially nonmetastatic (M0) CRC-PR patients. Across all 3 M0 cohorts, the TRS exhibited a significant stepwise escalation alongside advancing TNM/American Joint Committee on Cancer stages (Stages I to III) (Kruskal–Wallis test, all *P* < 0.05), supported by significant monotonic increasing trends (Spearman correlation, all *P* < 0.05) (Fig. S3A, C, and E). This correlation suggests that the TRS captures a dynamic prometastatic propensity accumulating within the primary TME prior to overt systemic spread.

Subsequently, Kaplan–Meier survival analyses stratified by optimal TRS cutoffs demonstrated that high TRS was significantly associated with accelerated disease progression. Specifically, elevated TRS correlated with shorter recurrence-free survival in the GSE39582 cohort (log-rank *P* = 0.0023), shorter disease-free survival in the GSE17536 cohort (log-rank *P* = 0.0004), and reduced progression-free survival in the TCGA-COADREAD cohort (log-rank *P* = 0.021) (Fig. S3B, D, and F). Collectively, these longitudinal results indicate that the model-derived TRS enables robust prospective risk stratification, identifying high-risk patients prone to future relapse prior to clinically established distant metastasis.

### Identification of core metabolic features driven by TRS

**Fig. 3.**
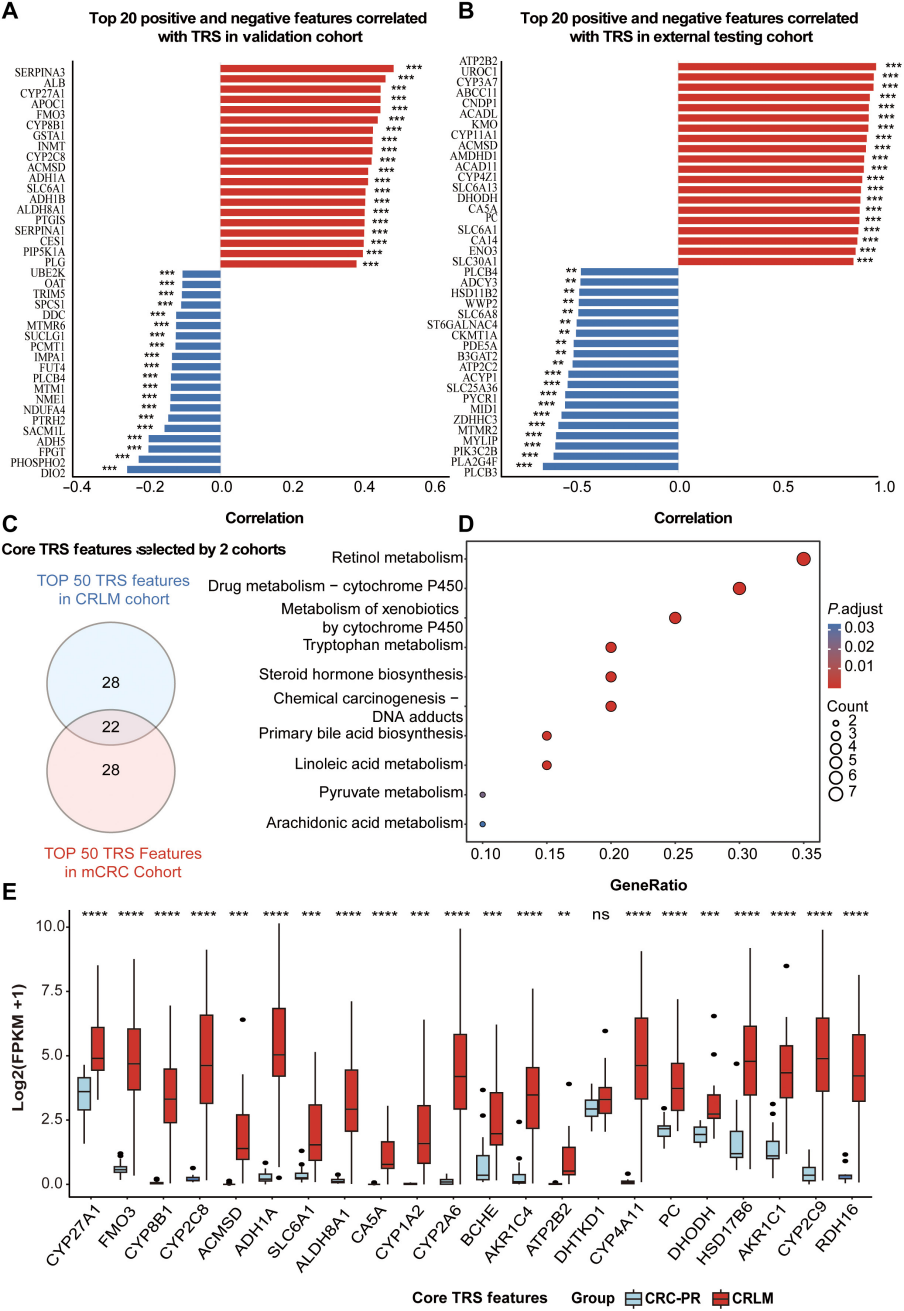
Identification and functional analysis of features most strongly correlated with the transcriptomic risk score (TRS). (A) Top 20 features showing the strongest positive or negative correlations with TRS in the combined metastatic colorectal cancer (mCRC) and primary colorectal cancer (CRC-PR) cohort (training and internal validation set). (B) Top 20 features showing the strongest positive or negative correlations with TRS in the external test cohort (CRLM and CRC-PR). Statistical significance (A and B) were evaluated using Spearman correlation. (C) Intersection of the top 50 TRS-associated features identified in (A) and (B), yielding 22 core TRS features. (D) Pathway enrichment analysis of the 22 core TRS features, highlighting significantly enriched metabolic and signaling pathways. (E) Differential expression analysis of the core TRS features between CRLM and CRC-PR samples in the GSE50760 dataset, presented as log2(FPKM+1). Red boxes indicate CRLM samples and gray boxes indicate CRC-PR samples; significance levels are marked with asterisks. Statistical differences between groups were assessed using a *t* test. **P < 0.01; ***P < 0.001; ****P < 0.0001; ns, not significant.

Based on the validated predictive capacity of the TRS system, feature selection was performed independently in the mCRC and CRLM cohorts to identify stable drivers of metastasis. In the mCRC cohort, the 20 features most strongly correlated with TRS were identified (Fig. 3A and Table S11). Similarly, the top 20 features in the CRLM cohort were extracted (Fig. 3B and Table S12). The intersection of the top 50 TRS-associated features from both cohorts yielded 22 core features closely linked to CRC metastasis risk (Fig. 3C and Table S13).

Notably, several of these 22 core features have established roles in CRC malignancy. For instance, SERPINA1 expression is correlated with poor prognosis and promotes migration via STAT3 activation [23], while PYCR1 facilitates liver metastasis through gut microbiota modulation [24]. DHODH supports metastatic growth by sustaining pyrimidine biosynthesis [25], and genes such as PTGIS [26], *PC* [27], and *SERPINA3* [28] have also been linked to metastatic progression. KEGG pathway enrichment analysis of these 22 features revealed significant involvement in multiple metabolism-related processes, including steroid hormone biosynthesis and tryptophan metabolism (Fig. 3D and Table S14). Furthermore, differential expression analysis confirmed that most of these core features were significantly up-regulated in CRLM tissues compared with CRC-PR tissues (*P* < 0.05; Fig. 3E). These findings reinforce the biological plausibility of the TRS core features as key molecular drivers of CRC metastatic development.

### Integrated multi-omics identifies core metabolic pathways in CRLM

To investigate key metabolic alterations in CRLM, serum from 15 patients with CRLM and 15 patients with localized CRC (LCRC) (June to October 2024; Sun Yat-sen Memorial Hospital) underwent untargeted LC-MS metabolomic profiling (Fig. S4A).

The OPLS-DA permutation testing reported the model’s excellent explanatory capacity (*R*^2^*Y* = 0.97) and predictive performance (*Q*^2^ = 0.72), both of which demonstrated highly significant statistical *P* values (Fig. S4B). PLS-DA clearly separated the CRLM and LCRC samples, indicating distinct metabolic signatures (Fig. S4C). DEM analysis revealed 139 significantly altered metabolites, of which 88 were up-regulated and 51 were down-regulated in CRLM (Fig. S4D).

Integration of the metabolomic and transcriptomic information was performed by comparing the KEGG pathway enrichment of the DEMs with the TRS core features (*n* = 22). Overlap analysis revealed 2 pathways that were significantly enriched in both datasets: retinol metabolism and tryptophan metabolism (Fig. S4E and Table S15). Integrated multi-omics enrichment confirmed the significant representation of these pathways in terms of both the metabolome and TRS-derived transcriptomic features (Fig. S4F), suggesting their involvement in CRLM progression.

GSEA further revealed significant up-regulation of both core pathways in CRLM tissues: retinol metabolism (normalized enrichment score = 2.46, adjusted *P* value < 0.001; Fig. S4G) and tryptophan metabolism (normalized enrichment score = 2.24, adjusted *P* value < 0.001; Fig. S4H). Collectively, these multi-omics analyses revealed distinct CRLM-associated metabolic alterations, identifying retinol and tryptophan metabolism as key pathways driving metastatic progression and providing leads for mechanistic and therapeutic exploration.

### A promising diagnostic biomarker identified from the CRLM metabolic network

**Fig. 4.**
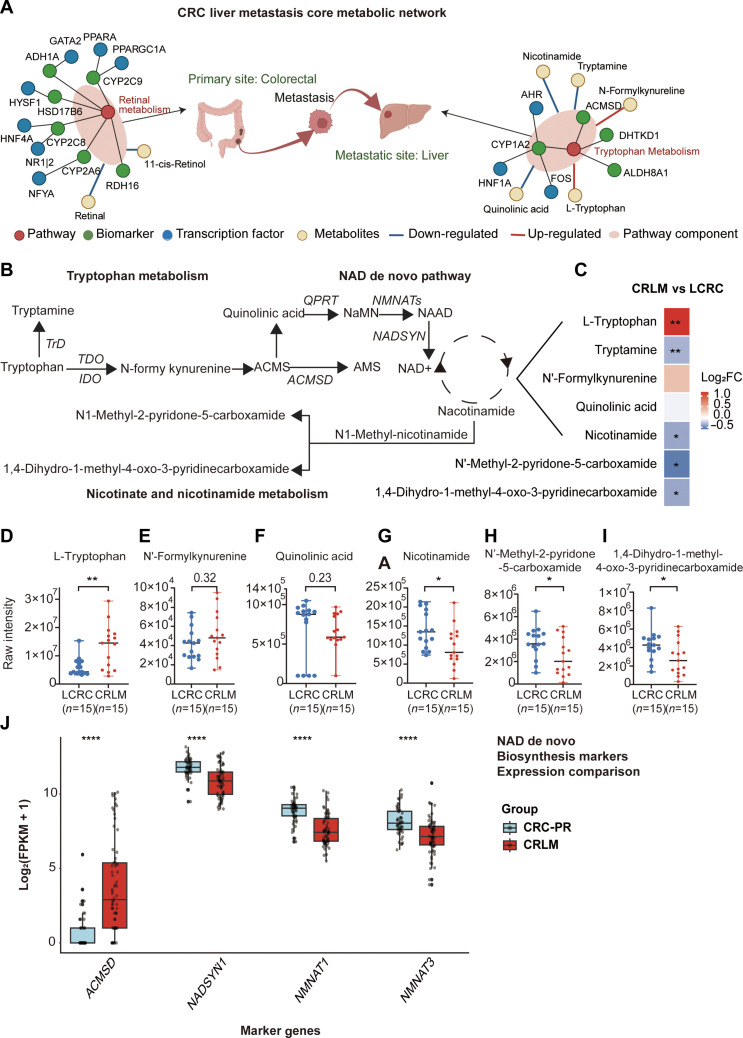
ACMSD as a diagnostic biomarker for CRLM and its involvement in nicotinamide adenine dinucleotide (NAD^+^) de novo pathway. (A) Integrated metabolomic–transcriptomic network for colorectal cancer liver metastasis (CRLM), incorporates key pathways, regulatory gene biomarkers, metabolites, and transcription factors. (B) Schematic diagram of the NAD^+^ de novo biosynthesis pathway highlighting the metabolic steps mediated by ACMSD within the tryptophan–quinolinate–nicotinate/nicotinamide axis. (C) Heatmap displaying the log_2_ fold change (log_2_FC) of key metabolites in the NAD de novo pathway between CRLM and localized CRC (LCRC) cohorts. (D to I) Scatter plots showing the raw intensity values for representative metabolites between CRLM and LCRC cohorts. Statistical significance was assessed using the Wilcoxon test. *P < 0.05; **P < 0.01;. (J) Box plots comparing the transcript levels (log_2_[FPKM+1]) of representative genes involved in NAD de novo biosynthesis (including ACMSD, NADSYN1, NMNAT2, and QPRT) between CRLM and prmary colorectal cancer (CRC-PR) cohorts (GSE204805). Statistical significance was determined by *t* test. ****P < 0.0001.

To elucidate the synergistic interplay between transcriptome and metabolomic alterations in CRLM, metabolic pathways enriched by DEMs were intersected with those enriched by TRS core features (*n* = 22). This integration established a CRLM core metabolic network encompassing key pathways, regulatory gene biomarkers, transcription factors, and metabolites (Fig. 4A). An evaluation of network-derived gene biomarkers against nonmetastatic CRC-PR tissues (Fig. S5A) revealed that *ACMSD* was the most promising diagnostic biomarker for CRLM, with the highest AUC. Notably, *ACMSD* has not previously been applied or extensively investigated for mCRC or CRLM screening, underscoring its novelty.

Benchmarking *ACMSD* against multiple previously reported CRLM biomarkers (Fig. S5B) confirmed the consistently favorable classification accuracy in distinguishing CRLM from CRC-PR samples, supporting its potential as an early screening and diagnostic tool. Clinicopathological analysis of the TCGA CRC cohort revealed that *ACMSD* expression was significantly positively correlated with pathological T stage and American Joint Committee on Cancer stage (Fig. S5C and D). Prognostic analysis further indicated that high *ACMSD* expression was associated with markedly reduced overall survival, with a pronounced decrease in survival probability within 5 years (Fig. S5E and F), suggesting that *ACMSD* could serve as an adverse prognostic factor in CRC.

### ACMSD reprograms tryptophan–NAD^+^ metabolism to impair NAD^+^ biosynthesis in CRLM

From a metabolomics perspective, the analysis focused on the tryptophan metabolism pathway in which ACMSD is located. ACMSD decarboxylates ACMS to picolinic acid, thereby reducing quinolinate levels in the bloodstream [29] (Fig. 4B). Consistent with this mechanism, serum from patients with CRLM exhibited impaired de novo NAD^+^ synthesis, which was characterized by reduced quinolinate and elevated upstream intermediates, including tryptophan and N-formyl kynurenine (Fig. 4C). These findings suggested decreased NAD^+^ biosynthesis efficiency, potentially attributable to ACMSD activity. Furthermore, serum nicotinamide [30] and NAD^+^ catabolites, including N-methyl-2-pyridone-5-carboxamide (N-Me-2PY) and N-methyl-4-pyridone-3-carboxamide (N-Me-4PY) [31], were significantly reduced in patients with CRLM (Fig. 4D to I), indicating decreased NAD^+^ turnover, consistent with transcriptomic data showing NAD^+^ synthesis insufficiency.

From a transcriptomic standpoint, high ACMSD expression in CRLM tissues was accompanied by coordinated down-regulation of key enzymes in the de novo NAD^+^ biosynthesis pathway, including NADSYN1, NMNAT1, and NMNAT3 (Fig. 4J). These findings suggested that increased ACMSD expression might impair NAD^+^ production by reducing substrate availability.

Collectively, the results of integrated multi-omics analyses indicated that ACMSD reprogrammed tryptophan metabolism to limit de novo NAD^+^ biosynthesis, thereby altering the energy metabolic state in patients with CRLM.

### ACMSD drives CRC progression and metastasis via the TGF-β/EMT pathway

To assess the clinical utility of ACMSD as a tissue detection biomarker, surgically resected tissue specimens were prospectively collected from 100 patients with CRC at Sun Yat-sen Memorial Hospital between January 2019 and June 2024. Patients were stratified into CRLM (*n* = 48) and LCRC (*n* = 52) cohorts, comprising 57 males and 43 females (mean age = 62 years; range = 26 to 88 years). Primary tumors were located in the colon in 79 patients and in the rectum in 21 patients. Baseline clinicopathological characteristics are summarized in Table 1.

**Table 1.** Baseline characteristics of enrolled patients. *P* values comparing patients in the 2 cohorts. *t* Test was used for all continuous variables. Fisher’s exact test was used for all categorical variables. The clinicopathological metrics include clinical metrics such as gender, age, and the primary tumor site, as well as pathological assessment metrics, including mismatch repair (MMR) status, pathological M stage, pathological T stage, and the American Joint Committee on Cancer (AJCC) stage. These metrics are integrated with genomic mutation characteristics, therapeutic regimens, and postoperative survivals to provide a multidimensional assessment of patient profiles.

Clinicopathological metric	All patients (*N* = 100)	CRLM (*N* = 48)	LCRC (*N* = 52)	*P* value
Gender				0.55
Male	57	29	28	
Female	43	19	24	
Age (years)				0.07
<65	54	30	24	
≥65	46	18	28	
Site				0.81
Colon	79	37	42	
Rectum	21	11	10	
IHC staining status				2.56E−08
Positive	57	41	16	
Negative	43	7	36	
MMR type				0.03
MSS	91	46	45	
MSI-H	6	0	6	
Unknown	3	2	1	
Pathological M stage				9.97E−30
M1	48	48	0	
M0	52	0	52	
Pathological T stage				2.00E−03
T1	1	1	0	
T2	17	4	13	
T3	52	21	31	
T4	27	20	7	
Unknown	3	2	1	
Pathological N stage				5.50E−04
NX	4	2	2	
N0	20	2	18	
N1a-1c	65	36	29	
N2a-2c	8	6	2	
Unknown	3	2	1	
Clinical stage				3.22E−07
≤II	19	0	19	
>II	80	48	32	
Unknown	1	0	1	
Mutation status				0.82
Mutant	24	13	11	
WT/Unknown	76	38	38	
Drug treatment				1.07E−29
Chemotherapy	21	21	0	
Target + chemotherapy	24	24	0	
PD-1 + chemotherapy	3	3	0	
Unknown	52	0	52	
Postsurgery prognosis				2.94E−04
Positive	12	6	6	
Negative	18	16	2	
Unknown	70	26	44	

IHC, immunohistochemical; LCRC, localized colorectal cancer; CRLM, colorectal liver metastasis; WT, wild type; MSS, microsatellite-stable; MSI-H, microsatellite instability-high; PD-1, programmed cell death protein 1

**Fig. 5.**
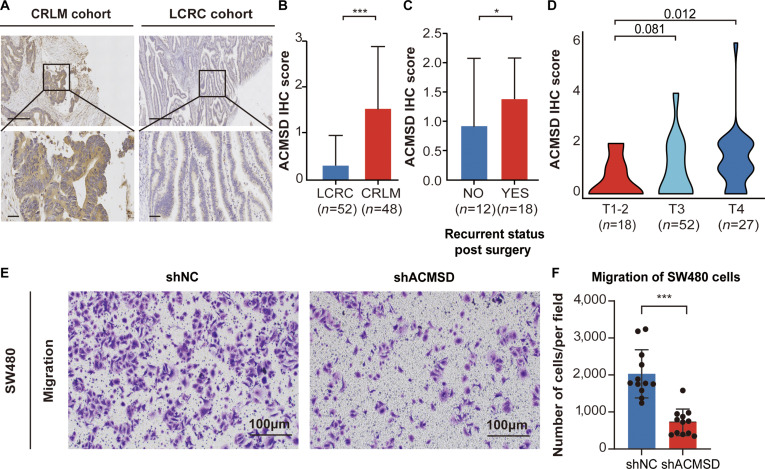
Clinical immunohistochemical (IHC) validation and in vitro assessment of the promigratory role of ACMSD in colorectal cancer (CRC). (A) Representative IHC staining images of ACMSD in CRC tumors from CRLM and localized CRC (LCRC) cohorts. Scale bars: 200 μm (10×), 50 μm (40×). (B) Quantified IHC scoring comparing ACMSD protein expression between CRLM and LCRC cohorts. (C) Quantified IHC scoring comparing ACMSD protein expression between recurrent CRC patients and nonrecurrent patients. (D) Violin plot showing the correlation between ACMSD protein expression (quantified IHC scoring) and pathological tumor stages (T1 to T2, T3, and T4). (E) Representative images of Transwell migration assays for SW480 cells transfected with either scramble small hairpin RNA (shRNA) (shNC) or ACMSD-targeting shRNA (shACMSD). Migrated cells are stained purple. Scale bar, 100 μm. (F) Quantification of migrated SW480 cells from Transwell assays. The graph shows the number of cells per field for shACMSD and shNC groups (*n* = 3 per group). Data points (dots) represent individual, nonoverlapping fields collected from 3 independent experiments. Statistical significance was determined by Wilcoxon rank-sum test. *P < 0.05; ***P < 0.001.

IHC staining (Table S16) revealed positive ACMSD signals in more than 70% of the CRLM specimens. Compared with the LCRC samples, the ACMSD samples displayed markedly stronger staining intensity in the CRLM tissue samples (Fig. 5A). Quantitative analysis further supported these observations, as the ACMSD protein expression level was significantly greater in the CRLM cohort (Fig. 5B). These results underscored that ACMSD served as a strong candidate tissue-based diagnostic biomarker for CRLM.

ACMSD protein expression was also significantly elevated in postoperative recurrent patients with CRC compared with that in recurrence-free individuals (Fig. 5C and Table S17). Within the CRLM cohort, high ACMSD expression correlated with an increased risk of postoperative recurrence, reinforcing its prognostic relevance. Analysis across pathological tumor stages (pT1 to pT4) revealed a stepwise increase in ACMSD protein abundance with increasing tumor invasiveness (Fig. 5D), indicating a positive correlation with tumor progression.

To elucidate the functional role of ACMSD in CRC cell migration, Transwell migration assays were performed using SW480 cells transfected with either control shRNA (shNC) or ACMSD-targeting shRNA (shACMSD). Compared with the shNC group, knockdown of ACMSD markedly reduced the number of migrating cells (Fig. 5E). Quantitative analysis confirmed a significant decrease in the number of migrated cells in the shACMSD group compared with that in the control group (Fig. 5F), indicating that ACMSD positively regulated the migratory capacity of CRC cells.

To further investigate the molecular mechanisms underlying the promigratory role of ACMSD in CRC, we knocked down ACMSD expression in NCI-H716 cells using shRNA, and the results revealed a greater than 50% reduction (Fig. S6A). RNA-seq analysis revealed that ACMSD knockdown significantly suppressed the activity of the epithelial-to-mesenchymal transition (EMT) pathway, a critical process for tumor metastasis [32] (Fig. S6B). This phenomenon was accompanied by decreased expression of key EMT signature markers (Fig. S6C), which collectively indicated clear inhibition of EMT signaling [33]. Given that EMT morphological changes are largely influenced by the TGF-β signaling pathway [33,34], we also observed significant down-regulation of the expression of the TGF-β pathway (Fig. S6D) and its signature markers upon ACMSD knockdown (Fig. S6E). These findings suggested that ACMSD promoted CRC cell migration by activating the TGF-β pathway, which subsequently induced EMT.

Collectively, these clinical validation data and mechanistic insights established ACMSD as a promising biomarker for CRLM prediction, with potential applications in both diagnosis and postoperative risk stratification. They further provided a mechanistic basis for its role in promoting CRC cell migration via TGF-β/EMT signaling.

### ACMSD as an indicator of immune infiltration and immunotherapy potential in patients with CRLM

**Fig. 6.**
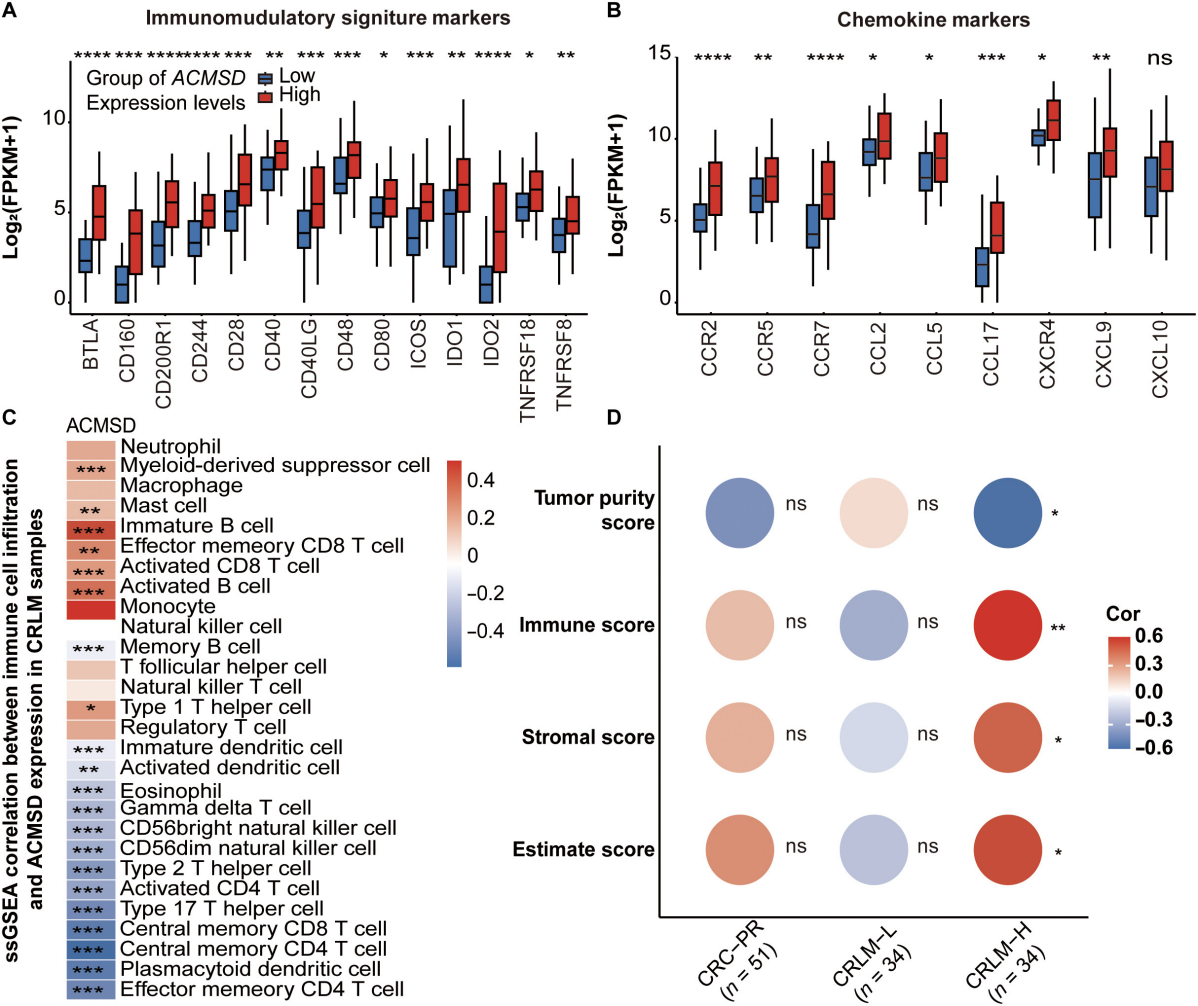
Association of ACMSD expression with immune cell infiltration and tumor microenvironment (TME) complexity in CRLM. (A) Comparative analysis of immunomodulatory signature marker gene expression between CRLM patients with high and low ACMSD expression. (B) Comparative analysis of chemokine marker gene expression between CRLM patients with high and low ACMSD expression. (C) Heatmap of single-sample gene set enrichment analysis (ssGSEA) results showing correlations between ACMSD expression and immune cell infiltration across multiple immune cell types in CRLM samples. Empty cells indicate nonsignificant associations. Statistical significance was determined using Pearson correlation. (D) Correlation between ACMSD expression and ESTIMATE scores, including tumor purity score, immune score, stromal score, and overall estimate score, across 3 groups: primary colorectal cancer (CRC-PR), CRLM with high ACMSD expression, and CRLM with low ACMSD expression. Statistical significance was determined using Spearman correlation. Statistical significance between groups was determined by *t* test. *P < 0.05; **P < 0.01; ***P < 0.001; ****P < 0.0001; ns, not significant.

In patients with CRLM, high ACMSD expression was associated with significantly elevated immunomodulatory (Fig. 6A) and chemokine (Fig. 6B) gene signatures, suggesting enhanced immune cell recruitment and inflammatory activation. ssGSEA revealed that ACMSD expression was positively correlated with increased TME complexity (Fig. 6C and Table S18), encompassing both immune-activating subsets (e.g., CD8^+^ T cells and B cells) and immune-suppressive subsets (e.g., regulatory T cells and myeloid-derived suppressor cells). Patients with high ACMSD expression had significantly higher immune and ESTIMATE scores but lower tumor purity (Fig. 6D and Table S19), which indicated pronounced immune infiltration.

GSEA further confirmed that high ACMSD expression was associated with the up-regulation of immune effector pathways, including T-cell receptor signaling (Fig. S7A) and natural killer cell-mediated cytotoxicity (Fig. S7B). Elevated ACMSD levels also strongly correlated with increased transcript abundance of immune checkpoint genes, including *PDCD1* (Fig. S7C), *CTLA4* (Fig. S7D), *CD274* (PD-L1) (Fig. S7E), *TIGIT* (Fig. S7F), *LAG3* (Fig. S7G), and *HAVCR2* (TIM-3) (Fig. S7H). This expression profile was characteristic of a “hot tumor” phenotype, suggesting heightened responsiveness to immune checkpoint inhibitors.

Leveraging the RNA-seq data from ACMSD-knockdown (shACMSD) and control (shNC) CRC cells, we further investigated the tumor-intrinsic immunomodulatory role of ACMSD. GSEA revealed that ACMSD knockdown significantly suppressed the activity of most proinflammatory and immune response signaling pathways (Fig. S8A). Consistently, the expression of key tumor-derived inflammatory and immunomodulatory genes markedly decreased in shACMSD cells (Fig. S8B), reflecting a weakened capacity of tumor cells to sustain an inflamed molecular environment. To further characterize these shifts, we utilized the Microenvironment Cell Populations-counter algorithm to evaluate the gene expression programs typically associated with immune recruitment [35]. We found that compared with shNC cells, shACMSD cells exhibited significantly lower scores for these immunomodulatory programs (Fig. S8C).

These findings suggested that tumor-intrinsic ACMSD loss reduced the number of molecular precursors necessary for immune recruitment, which logically aligned with the increased immune infiltration observed in high-ACMSD-expressing patient tissues. Notably, while actual immune cell infiltration occurred only within the complex TME of bulk tissues, these in vitro results provided biological support for the role of ACMSD in modulating the tumor–immune interface.

Collectively, these findings suggested that ACMSD served as a predictive indicator of an inflamed, immune-infiltrated marker with stronger immune responsive potential. These in vitro findings corroborated these clinical observations, demonstrating that ACMSD actively modulated immune-related pathways and immune cell infiltration. These findings underscore its potential value in assessing immunotypes and guiding immunotherapy strategies for CRLM patients.

### ACMSD is associated with drug strategy and therapeutic efficacy in CRLM patients

**Fig. 7.**
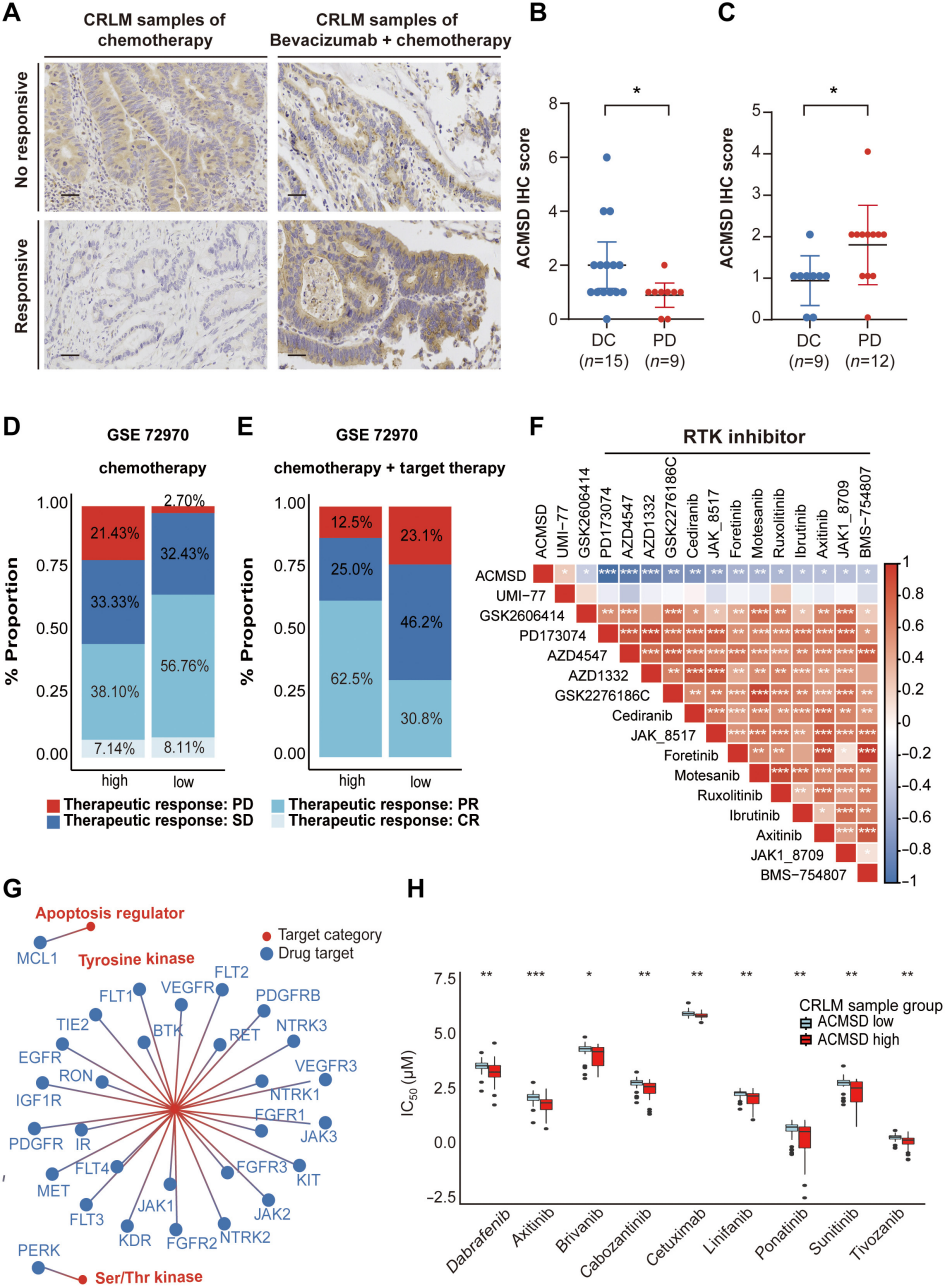
Clinical and therapeutic relevance of ACMSD as a diagnostic biomarker for CRLM and its association with drug sensitivity. (A) Representative immunohistochemical (IHC) images and quantification of ACMSD expression in CRLM samples, comparing patients with positive and negative responses to chemotherapy alone or in combination with bevacizumab. Scale bar = 50 μm (20×). (B and C) Comparison of ACMSD protein expression levels between disease control (DC) and progressive disease (PD) groups in CRLM patients receiving combined targeted therapy with chemotherapy (B) or chemotherapy alone (C). Statistical significance was assessed using the Wilcoxon test. *P < 0.05 . (D and E) Proportional distribution of therapeutic responses in CRLM patients stratified by high and low ACMSD expression levels, treated with chemotherapy alone (D) or chemotherapy plus bevacizumab and cetuximab (E), based on the GSE72970 dataset. (F) Heatmap showing drugs significantly correlated with ACMSD transcript levels in colorectal cancer (CRC) cell lines (*n* = 45) from the Genomics of Drug Sensitivity in Cancer (GDSC) database, with drug sensitivity represented by half-maximal inhibitory concentration (IC_50_) values. (G) Target interaction network of drugs identified in (F), where red nodes represent target categories and blue nodes represent individual drug targets. (H) Multivariate boxplots generated using OncoPredict showing predicted sensitivity to targeted therapies in CRLM patients with high or low ACMSD expression levels (GSE204805). Statistical significance was determined using the *t* test. *P < 0.05; **P < 0.01; ***P < 0.001.

Chemotherapy and targeted therapy (primarily EGFR/VEGFR inhibitors) remain the mainstay treatments for CRLM [3]. ACMSD expression patterns varied markedly by therapeutic modality. Responders to combined targeted therapy plus chemotherapy exhibited significantly higher ACMSD expression, whereas responders to chemotherapy alone presented lower levels (Fig. 7A). IHC further confirmed the elevated ACMSD protein abundance in patients who achieved disease control with combination therapy (Fig. 7B and Table S20), as compared with reduced levels in chemotherapy-alone disease control cases (Fig. 7C and Table S21).

In the chemotherapy-only group, 21.4% of patients with high ACMSD expression experienced progressive disease, as compared with only 2.7% of patients in the low-expression group (Fig. 7D). In contrast, in the combined therapy cohort, patients with high ACMSD expression exhibited markedly better outcomes, including a significantly lower incidence of progressive disease (Fig. 7E), suggesting a preferential benefit from targeted therapy in this subgroup.

Drug response profiling based on Genomics of Drug Sensitivity in Cancer (GDSC) data revealed compounds with inhibitory potency (half-maximal inhibitory concentration) that was significantly correlated with ACMSD transcript levels across 45 CRC cell lines (Fig. 7F and Table S22). Elevated ACMSD expression was strongly associated with increased sensitivity to multiple receptor tyrosine kinase (RTK) inhibitors, including VEGFR/EGFR inhibitors (vandetanib, gefitinib, lapatinib, and afatinib) and agents targeting downstream RTK signaling pathways (Table S23).

GDSC network mapping of ACMSD-associated compounds revealed that these drugs primarily targeted RTK regulators (Fig. 7G). VEGFR1/2/3 and EGFR emerged as central nodes, suggesting that ACMSD enhanced susceptibility to VEGFR/EGFR blockade. Using OncoPredict [36], drug response modeling of CRLM samples stratified by ACMSD expression further demonstrated that high-ACMSD cases were significantly more sensitive to VEGFR and EGFR inhibitors, including cetuximab and other clinically approved agents (Fig. 7H and Tables S24 and S25).

Collectively, these results suggested that high ACMSD expression in both mCRC cell lines and CRLM tissues implied limited benefit from chemotherapy alone but significant responsiveness to regimens incorporating EGFR/VEGFR-targeted agents. Altogether, these retrospective and computational associations suggest ACMSD as a valuable hypothesis-generating biomarker for evaluating drug responses. However, its definitive role in guiding personalized therapeutic strategies in CRLM management awaits prospective validation.

## Discussion

CRLM, the most common metastatic form of mCRC, has a poor prognosis, and early detection is crucial because of the lack of reliable screening biomarkers. Early diagnosis of mCRC substantially improves liver metastasis resectability and increases 5-year survival rates [37]. Current CRLM detection relies heavily on imaging modalities, including computed tomography, magnetic resonance imaging, positron emission tomography, and ultrasound, which achieve detection rates of only 50% to 70% [38]. These techniques provide limited predictive value for CRLM occurrence and offer minimal mechanistic insight [39]. Most established biomarkers, such as *KRAS/NRAS* and *BRAF* mutations, *UGT1A1* testing [40–42], or microsatellite status (microsatellite-stable/microsatellite instability) [43,44], primarily inform treatment response or prognosis after diagnosis rather than enabling early screening. General serum markers, such as carcinoembryonic antigen, carbohydrate antigen 19-9, and AFP, also lack specificity for CRLM [8,45].

Several candidate biomarkers for CRLM have been proposed, such as CXCL12/CXCR4, EGFR, COX2, CDX2, and HER-2; however, many require further validation and display limited predictive ability from primary tumor biopsies or are present at low prevalence [46–48]. These shortcomings reinforce the need for specific early-stage CRLM biomarkers. Traditional biomarker discovery strategies, often based on differential expression analyses or hypothesis-driven investigations, have proven valuable but face significant limitations in the current era of high-dimensional datasets. Such approaches remain inefficient for handling large-scale transcriptomic data and lack the capacity to integrate multi-omics profiles with clinical information across diverse patient cohorts. These constraints become increasingly evident as data complexity continues to expand [49,50].

Despite the substantial performance of the DeepMetabio-mCRC Screener in early metastatic risk prediction, we acknowledge that predicting postresection recurrence remains a critical clinical challenge. Our preliminary validation revealed that ACMSD protein expression was significantly elevated in patients who experienced postoperative recurrence compared with those who remained recurrence-free, supporting its inherent potential for predicting postoperative recurrence. Future research should leverage this framework to define biological, rather than merely technical, contraindications to surgery. Specifically, we aim to implement longitudinal tracking of postoperative cohorts by integrating the DeepMetabio framework with dynamic liquid biopsy data (e.g., circulating tumor DNA ) to capture “nonsalvageable” recurrences at their earliest stages.

Furthermore, the molecular mechanism by which ACMSD-mediated metabolic reprogramming drives relapse warrants further investigation. Accumulating evidence indicates that NAD^+^ metabolism is a pivotal driver of refractory disease and therapeutic resistance in CRC. Recent studies have demonstrated that nicotinamide phosphoribosyltransferase, the rate-limiting enzyme in NAD^+^ biosynthesis, promotes CRC cell proliferation and induces cancer stem cell-like properties through poly(ADP-ribose) polymerase and sirtuin 1 signaling, thereby enriching cancer-initiating cell populations [51,52]. Given that ACMSD diverts substrates away from de novo NAD^+^ biosynthesis, we hypothesize that ACMSD-driven metabolic reprogramming significantly modulates the energy homeostasis and stemness of the TME, contributing to postoperative relapse. Our future work will focus on integrating metabolite quantification with transcriptomic scoring to develop a “Metabolic-Recurrence Index”. Such a tool could enable clinicians to prioritize high-risk patients for intensive systemic therapy or dynamic surveillance over immediate surgical intervention.

While our multi-omics evidence is consistent with altered NAD^+^ metabolic flux mediated by ACMSD, we recognize the inherent complexity of tumor metabolism. Specifically, the NAD^+^ salvage pathway—which utilizes precursors like nicotinamide or nicotinamide riboside—represents a crucial compensatory mechanism in CRC. Although ACMSD diverts substrates from the de novo pathway, tumor cells may leverage salvage synthesis to maintain NAD^+^ homeostasis. Future studies utilizing isotopic metabolic flux analysis and direct enzymatic activity measurements are required to precisely define the net impact of ACMSD on total NAD^+^ pools.

While the DeepMetabio-mCRC Screener effectively identifies high-risk biological signatures associated with metastasis and enables robust longitudinal risk stratification, we acknowledge that its direct prospective predictive power requires careful contextualization. Although extensive recurrence-free, disease-free, and progression-free survival data from 3 independent cohorts strongly support the prospective stratification value of the TRS, pure “liver-metastasis-specific time-to-event” cohorts remain remarkably scarce in public databases. Consequently, the framework primarily discriminates metastatic transcriptomic states and infers metastatic propensity. Its direct capability to predict the precise timing of future metastasis to specific organs, such as the liver, remains partially inferential and necessitates further validation. Future efforts should prioritize large-scale, organ-specific, treatment-naïve longitudinal cohorts to definitively establish the accuracy and clinical utility of this framework in predicting future liver metastasis in initially non-mCRC patients.

The rigorous LODO cross-validation highlighted the complexities of applying a fixed binary threshold across highly heterogeneous cohorts. While demonstrating robust overall performance (average LODO AUC = 0.806, average PR-AUC = 0.752), noticeable performance fluctuations in specific cohorts like GSE81986 and COAD-SILU stemmed from threshold calibration artifacts and extreme class imbalance rather than biological feature extraction failures. Specifically, the GSE81986 cohort consists entirely of early-stage tumors with generally low absolute TRS probabilities; applying a rigid 0.5 threshold triggered a statistical collapse in binary metrics, even though the continuous TRS retains powerful relative prognostic value for longitudinal risk stratification. Similarly, COAD-SILU validation was confounded by extreme class imbalance (metastasis prevalence < 5.7%) and platform-induced domain shifts (microarray versus RNA-seq). These findings indicate that while the model effectively ranks metastatic risk, direct binary classification across diverse datasets necessitates cohort-specific threshold recalibration, emphasizing the utility of continuous risk stratification in future clinical applications.

Furthermore, although ACMSD expression levels significantly correlated with CRLM progression, ACMSD likely served as a surrogate marker for broader metabolic reprogramming within the tryptophan–NAD^+^ axis in CRLM rather than being the exclusive driver of these complex systemic alterations. Additionally, ACMSD should be characterized as a modulator of tumor-intrinsic immunomodulatory programs rather than a direct driver of systemic immune cell recruitment. While findings from cell-based assays suggest an influence on the molecular precursors of immune recruitment, literal immune infiltration claims remain restricted to bulk tumor datasets, and the specific mechanistic role of ACMSD in modulating the immune interface warrants further validation in coculture models.

Moreover, we explicitly acknowledge that our immune infiltration estimates, which were derived from bulk tumor datasets using ssGSEA and ESTIMATE algorithms, remain computational inferences rather than direct in vivo measurements. To definitively confirm the precise role of ACMSD in modulating the tumor–immune microenvironment, future orthogonal validations utilizing spatial transcriptomics, multiplex immunohistochemistry, or flow cytometry are strictly required. Similarly, our findings concerning heightened sensitivity to EGFR/VEGFR inhibitors in patients with high ACMSD expression rely primarily on computational modeling (e.g., GDSC and OncoPredict) and retrospective associations. Therefore, these therapeutic insights should be interpreted as valuable hypothesis-generating associations rather than prospectively validated predictive biomarkers. Prospective clinical validation in randomized trials remains strictly necessary before these insights can be translated into established therapeutic decision-support tools.

In addition, we acknowledge that the serum metabolomics cohort (*n* = 30) was designed as a discovery-phase investigation. Its relatively small sample size may limit the statistical power for certain downstream metabolites, which could affect the robustness of individual metabolite-level conclusions when applied to highly heterogeneous populations. Consequently, we have rigorously reframed these specific signals as “hypothesis-generating, pathway-level supportive evidence”, which more objectively reflects their scientific positioning within a small-sample exploratory cohort. To further mitigate the risk of false discovery, we substantiated the pathway-level metabolomic findings through orthogonal protein-level validation of the core regulatory enzyme, ACMSD, in an expanded clinical cohort of 100 patients. Nevertheless, future studies should prioritize larger, multicenter prospective cohorts and employ targeted absolute quantification assays to refine predictive thresholds and definitively establish the generalizability of these metabolic reprogramming signatures in CRLM screening.

To address these challenges and limitations, our study presents the DeepMetabio-mCRC Screener, an artificial intelligence (AI)-driven multi-omics framework that combines large-scale transcriptomics, deep learning, and serum metabolomics for early mCRC screening. We acknowledge that since the input gene vectors lack a natural spatial or biological ordering, the performance advantage of the 1D-CNN over classical tree-based models and fully connected DNNs likely stems from the convolutional inductive bias. Specifically, mechanisms such as parameter sharing and sparse connectivity provide strong implicit regularization, which effectively suppresses overfitting in high-dimensional transcriptomic spaces and enhances model robustness under distribution shifts.

Our framework resonates with recent breakthroughs in computational oncology. For instance, platforms like CRCDB and OligoM-Cancer highlight the power of multidimensional data integration for uncovering CRC pathogenesis and guiding precision metastasis management [53,54]. Algorithmically, our deep learning approach aligns with the growing trend of deploying advanced neural networks in CRC, such as the Gra-CRC-miRTar framework [55]. Furthermore, while our study focuses on transcriptomic and metabolic reprogramming, these dynamics are fundamentally linked to complex upstream epigenetic plasticity during CRC progression [56]. Future iterations of DeepMetabio could integrate these epigenetic and knowledge-guided dimensions to further refine holistic metastatic risk prediction

In addition to providing a new tool for CRLM detection, the present work serves as a proof of concept, establishing a systematic workflow for translating complex patient-derived omics data into clinically actionable insights. This workflow guides the development of highly accurate predictive models and the subsequent identification of biomarkers, such as ACMSD, that possess strong translational potential. The successful implementation of our framework highlights the significant potential of integrating multi-omics data to elucidate complex disease mechanisms. We envision that our screener and the biomarkers that it identifies will be incorporated into routine clinical practice in the future, enabling more precise patient stratification, guiding personalized therapeutic decisions, and ultimately improving outcomes for patients with CRC. We believe that this integrated workflow provides a versatile and effective paradigm that can be applied to a broader range of tumor studies, thereby accelerating the discovery of original solutions for precision medicine.

## Conclusion

In this study, we developed an AI-driven multi-omics framework that integrates large-scale transcriptomics, deep learning, and serum metabolomics for early mCRC screening. The DeepMetabio-mCRC Screener demonstrated high sensitivity and consistent generalizability in distinguishing mCRC from CRC-PR. By combining model-derived TRS with serum metabolomic profiling, a core metabolic network in CRLM was constructed, leading to the identification of ACMSD as a promising biomarker with strong diagnostic utility and clinical translational potential. Patient tissue validation, in vitro experiments, and bioinformatics correlation analyses further revealed that ACMSD was associations strongly associated with adverse prognosis, the reprogramming of NAD^+^, the modulation of the tumor immune microenvironment, and drug therapeutic responsiveness. Collectively, these findings underscore the potential of AI-driven multi-omics frameworks to advance early cancer detection, accelerate biomarker discovery, and provide actionable insights for personalized CRLM management.

### Key points

• The DeepMetabio-mCRC Screener, a 1D CNN integrating multicohort transcriptomic profiles, was developed for substantial early detection of mCRC.
• Model-derived TRSs revealed 22 core metabolic features enriched in retinol and tryptophan metabolism, highlighting key pathways involved in CRLM.
• Cross-omics integration revealed ACMSD as a diagnostic and prognostic biomarker that mechanistically impaired NAD^+^ biosynthesis and the immune-inflamed TME.
• Clinical and in vitro validation demonstrated that the ACMSD could predict CRLM risk, postoperative recurrence, metastatic mechanisms, and therapeutic responsiveness to EGFR/VEGFR-targeted agents, supporting its application in personalized treatment strategies.

## Ethical Approval

This study was conducted in accordance with the Declaration of Helsinki (revised in 2013) and was approved by the ethics committee of Sun Yat-sen Memorial Hospital (number SYSKY-2024-704-01).

## Data Availability

The data used in this study are available from the following public repositories: the Genomics of Drug Sensitivity in Cancer (GDSC) database (https://www.cancerrxgene.org/), the GEO database (https://www.ncbi.nlm.nih.gov/geo/), and the TCGA and COAD-SILU databases (https://www.cbioportal.org/). The source code for the DeepMetabio-mCRC Screener is available at: https://github.com/vivianne001/DeepMetabio-mCRC-A-Multi-Omics-Framework-for-Early-Prediction-of-Colorectal-Cancer-Liver-Metastasis. The sequencing data generated during this study are also available from the corresponding authors upon reasonable request.
