# Supplementary material for: DeepMetabio-mCRC Screener: A Multi-Omics Deep Learning Framework for Early Risk Prediction and Biomarker Discovery in Colorectal Liver Metastasis
Source: Comput Struct Biotechnol J. 2026 May 25;35(1):0074. doi: 10.34133/csbj.0074 (PMC13199651; doi:10.34133/csbj.0074)
Supplement: Supplementary 1 — Figs. S1 to S8 Tables S1 to S27 [file csbj.0074.f1.zip › Supplementary Legends.docx]

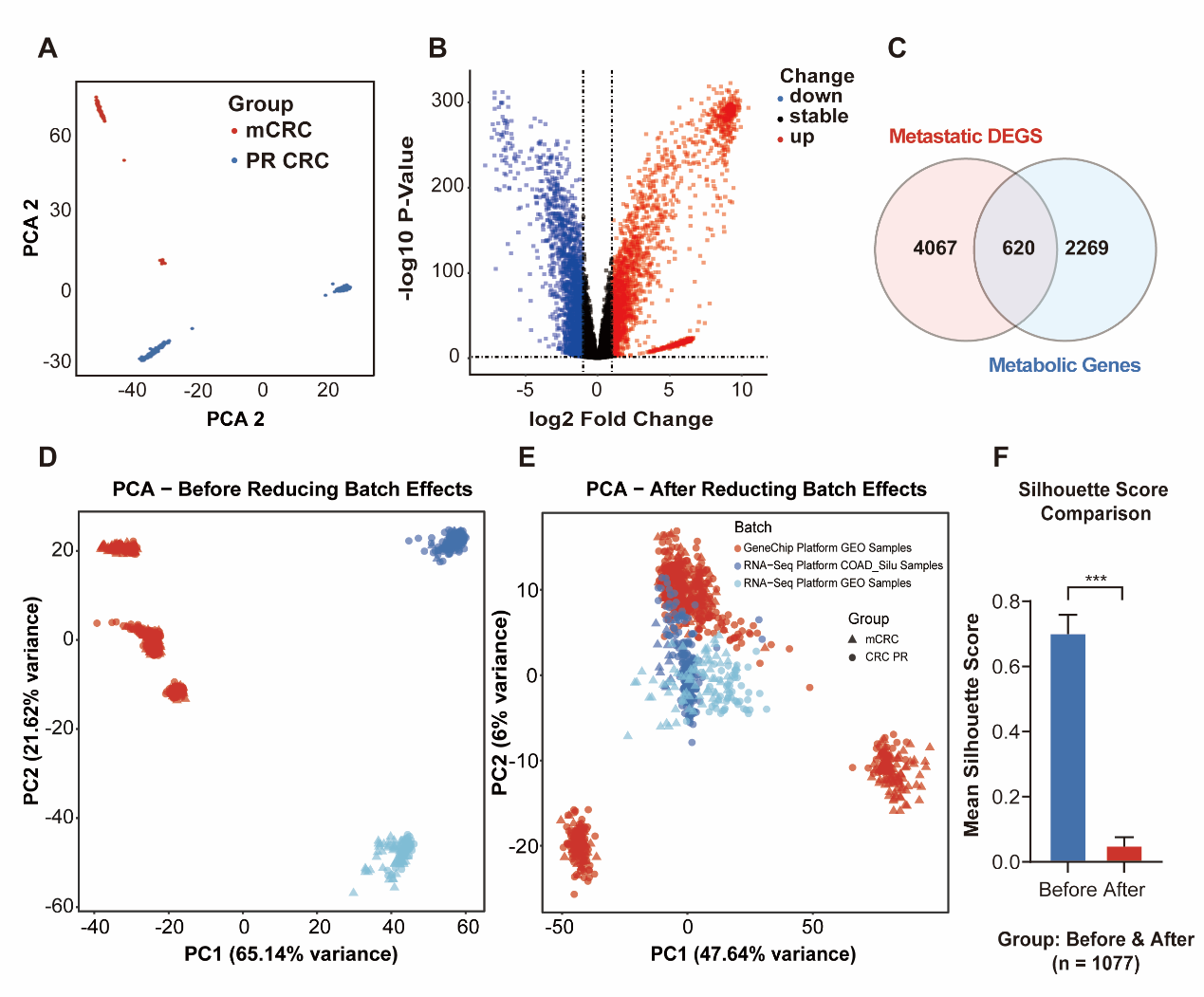


**Figure S1. Preprocessing of candidate biomarkers as inputs for the mCRC prediction model.**
(A) Principal component analysis (PCA) of metastatic colorectal cancer (mCRC) and primary colorectal cancer (CRC PR) groups in the GSE131418 dataset.
(B) Volcano plot of differentially expressed genes (DEGs) between mCRC and CRC PR. Genes with |log₂ fold change| > 1 and *P* < 0.05 were considered as DEGs, resulting in a total of 4,067 mCRC-associated DEGs identified from GSE131418.
(C) Venn diagram showing the intersection between DEGs and a predefined metabolic gene set across 32 human cancers, yielding 620 functional DEGs.
(D,E) PCA plots of cross‑platform samples before (D) and after (E) ComBat batch effect correction. (F) Comparison of mean silhouette scores before and after batch effect correction to evaluate clustering tendency. Lower silhouette scores after correction indicate effective reduction of batch effects. Statistical differences between groups were assessed using a t-test. **P* < 0.05; ***P* < 0.01; ****P* < 0.001; ns, not significant.


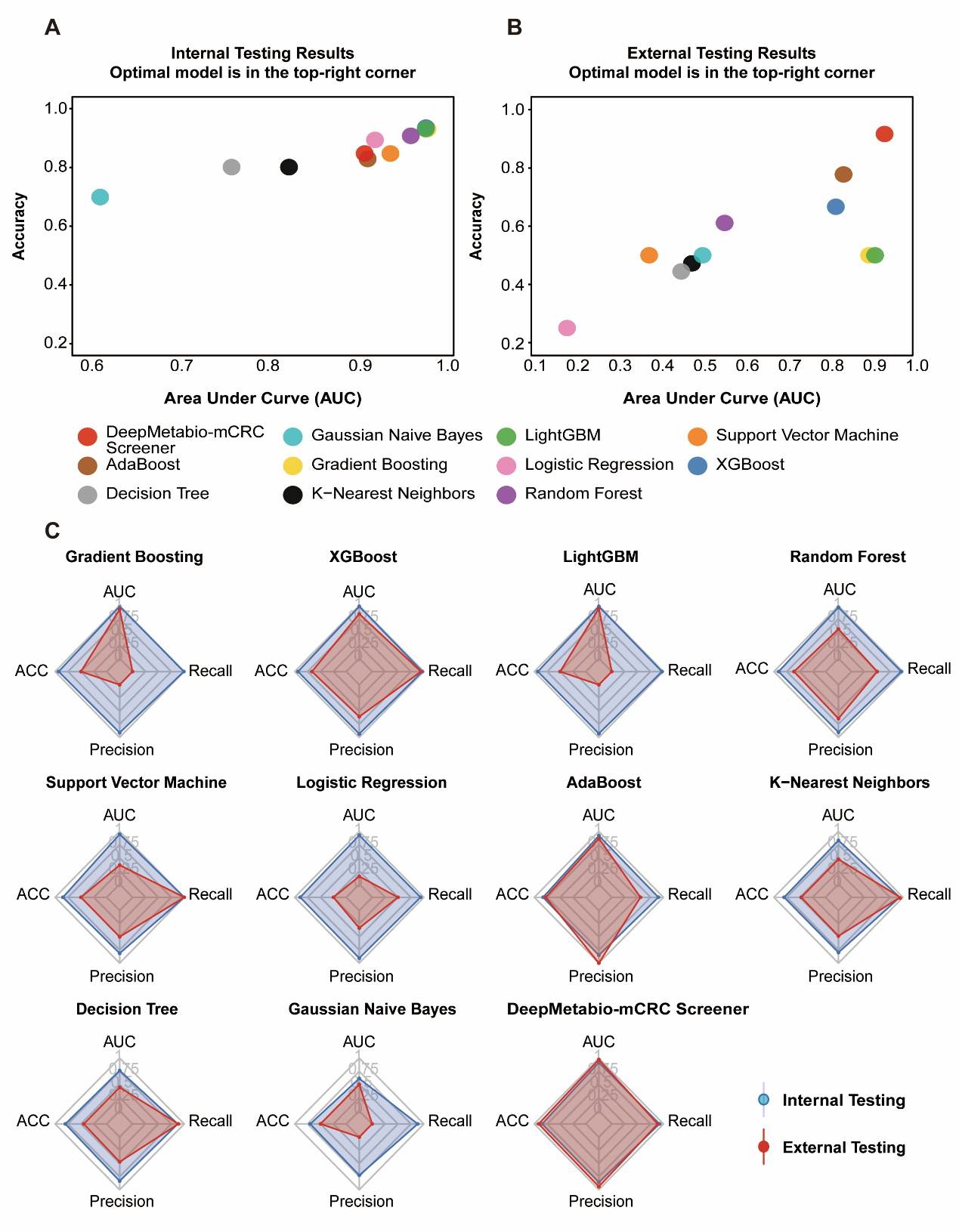


**Figure S2. Benchmark testing of the DeepMetabio‑mCRC Screener against 10 classical machine learning models.**

(A) Comparison of classification performance on the internal validation set, with the x‑axis representing AUROC and the y‑axis representing accuracy. Models positioned in the upper right quadrant indicate optimal performance.

(B) Comparison of classification performance on the external test set, with the x‑axis representing AUROC and the y‑axis representing accuracy. Models in the upper right quadrant indicate optimal performance.

(C) Radar plots illustrating accuracy, AUROC, precision, and recall for each model on both the internal validation set and the external test set. Blue lines indicate internal validation performance, and orange lines indicate external test performance.


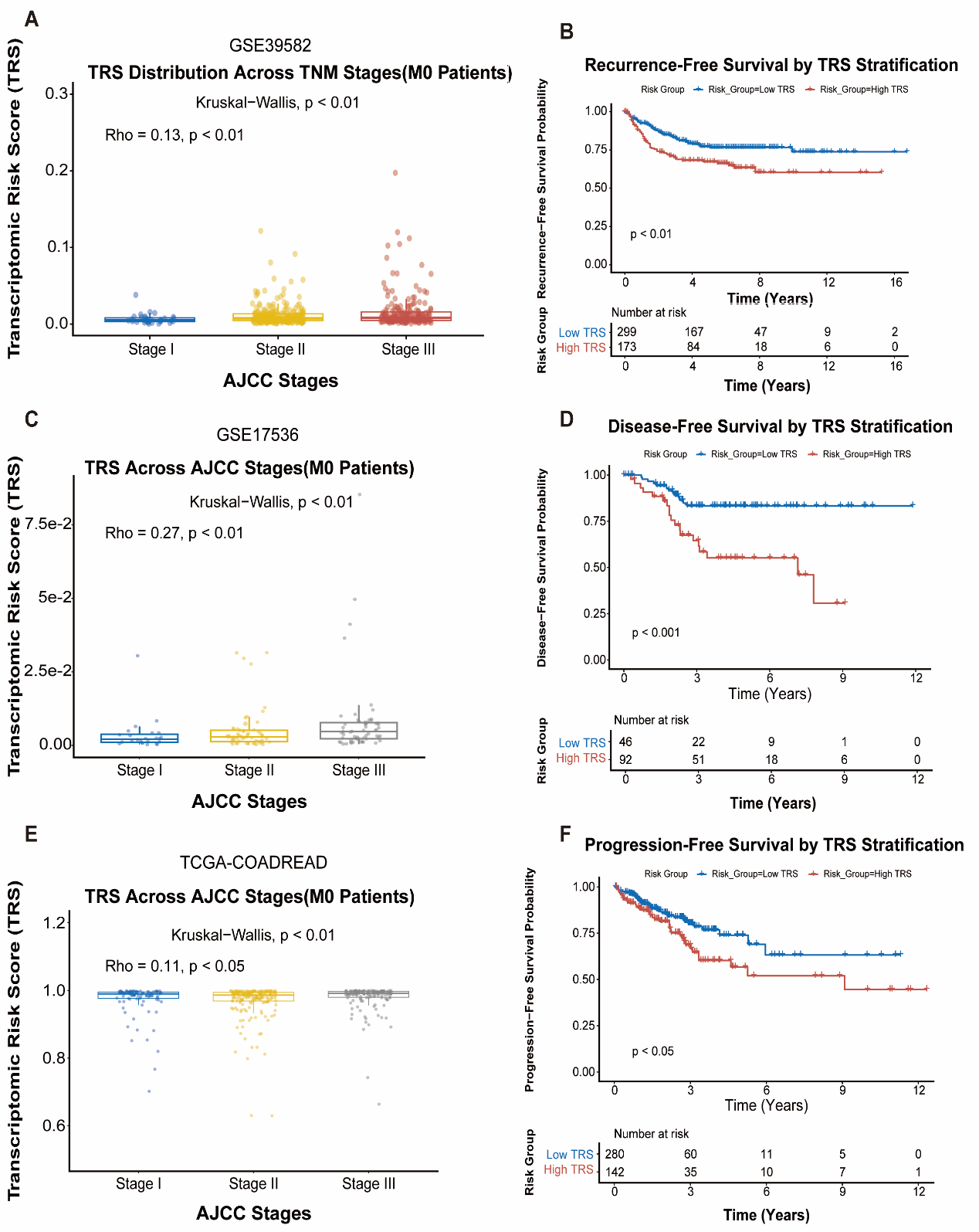


**Figure S3. Prospective validation of the Transcriptomic Risk Score (TRS) for longitudinal risk stratification and its association with disease progression in three independent initially non-metastatic (M0) cohorts.**

(A, C, E) Boxplots illustrating the horizontal molecular evolution of TRS across localized clinical stages (AJCC Stage I-III) in the GSE39582 (A), GSE17536 (C), and TCGA-COADREAD (E) cohorts. All patients presenting with established distant metastasis at diagnosis (Stage IV/M1) were strictly excluded to ensure a genuine assessment of initially non-metastatic populations. Statistical significance measuring the progressive escalation of TRS and tumor advancement were calculated by Kruskal-Wallis. Rho ranging was calculated by Spearman’s correlation. (B, D, F) Kaplan-Meier survival curves demonstrating the robust longitudinal risk stratification capacity of TRS. Despite the absence of distant metastasis at diagnosis, primary M0 patients with higher TRS exhibited a significantly shorter Recurrence-Free Survival (RFS) in GSE39582 (B), Disease-Free Survival (DFS) in GSE17536 (D), and Progression-Free Survival (PFS) in TCGA-COADREAD (F).


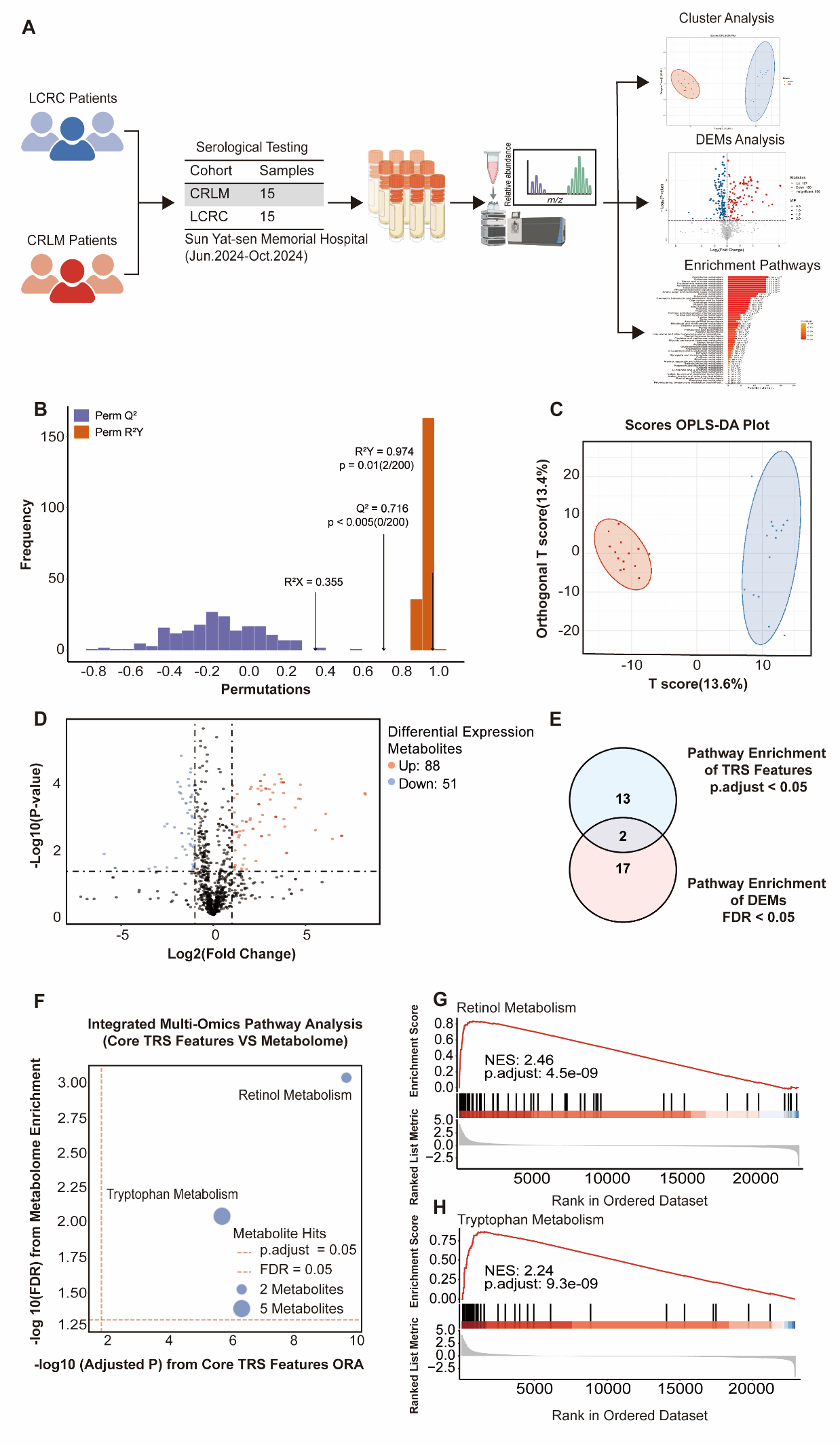


**Figure S4. Robust metabolomics profiling and integrated multi-omics pathway analysis to identify key CRLM-associated metabolic alterations.**

(A) Workflow of metabolomics analysis: serum samples from patients with late‑stage colorectal cancer (LCRC, n = 15) and colorectal cancer liver metastasis (CRLM, n = 15) were collected and analyzed by LC-MS, followed by metabolite clustering, differential expression analysis, and pathway enrichment analysis.

(B) Validation of the OPLS-DA model using a 200-iteration permutation test. The histogram displays the distribution of permuted *R*^2^*Y* and *Q*^2^ values, demonstrating the original model's excellent explanatory capacity (*R*^2^*Y* = 0.974, p = 0.01) and predictive performance (*Q*^2^ = 0.716, p < 0.005) without evidence of overfitting.

(C) OPLS‑DA score plot showing clear separation between CRLM and LCRC samples.

(D) Volcano plot highlighting differentially expressed metabolites (DEMs) associated with CRLM. Metabolites were identified as DEMs based on a log_2_ fold change >1 or <−1 and *P* value < 0.05.

(E) Venn diagram illustrating the overlap between pathways enriched from TRS core features and those enriched from DEMs, with only pathways meeting *p*.adjust < 0.05 or FDR significance retained for intersection analysis.

(F) Integrated multi-omics pathway analysis scatter plot summarizing the intersecting pathways and their enrichment significance. Retinol metabolism and tryptophan metabolism emerged as the most representative enriched pathways across both transcriptomic and metabolomic dimensions.

(G, H) Gene set enrichment analysis (GSEA) curves of the retinol metabolism (G) and tryptophan metabolism (H) pathways in CRLM samples (GSE50760 dataset).


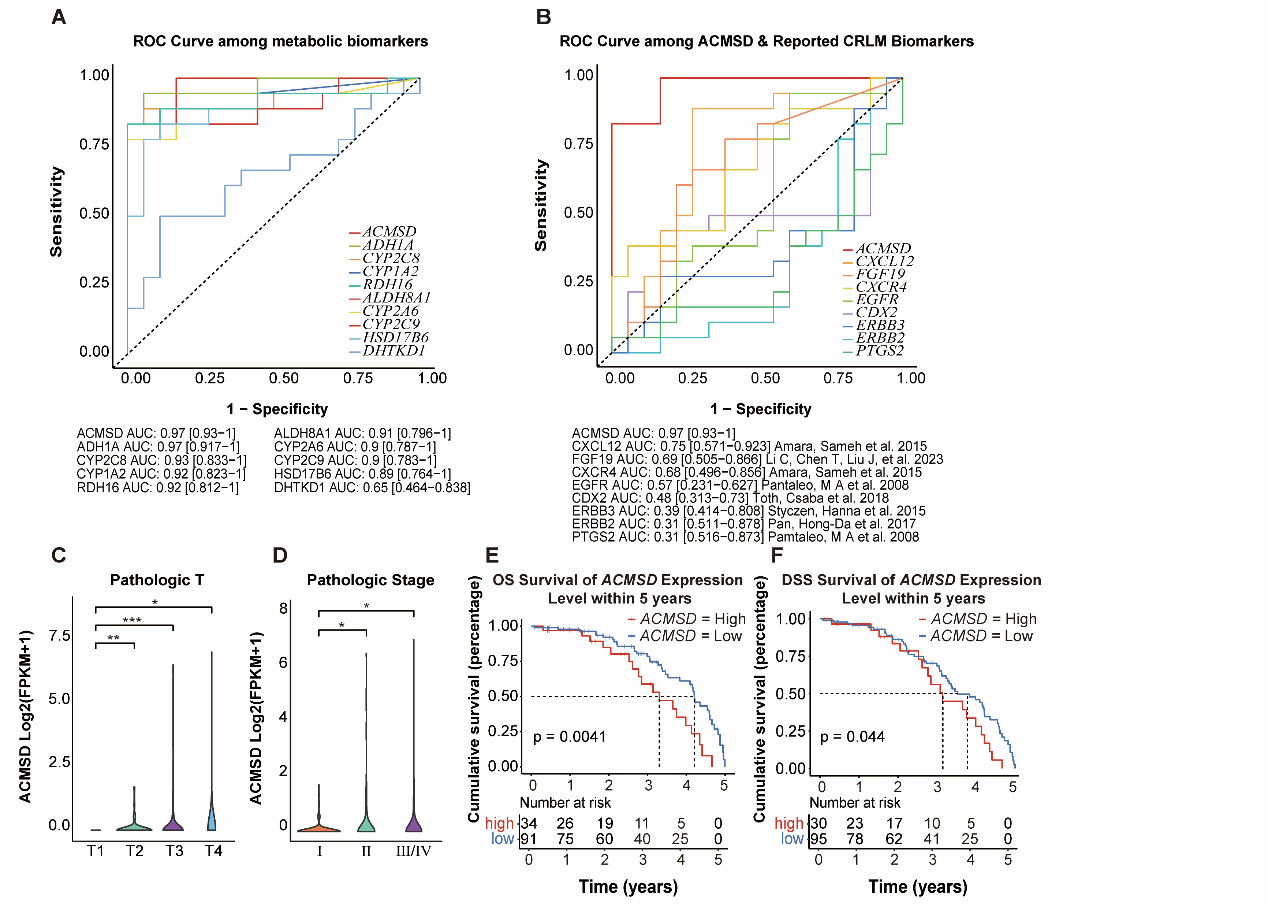


**Figure S5. Selection of the optimal CRLM predictor, and its association with CRC progression and prognosis.**

(A) ROC curves comparing ACMSD with TRS core features identified from the metabolic network as candidate biomarkers for distinguishing CRLM from CRC PR samples.

(B) ROC curves comparing ACMSD with previously reported CRLM predictive biomarkers in differentiating CRLM from CRC samples.

(C, D) Violin plots showing ACMSD expression levels in CRC samples from TCGA across increasing cancer severity, grouped by AJCC stage (C) and TNM system (D). Statistical differences between groups were assessed by t-test. **P* < 0.05; ***P* < 0.01; ****P* < 0.001; ns, not significant.

(E, F) Kaplan–Meier survival curves illustrating that both overall survival (OS) (E) and disease-specific survival (DSS) (F) were significantly shorter in the ACMSD-high group compared with the ACMSD-low group in the GSE17536 cohort.


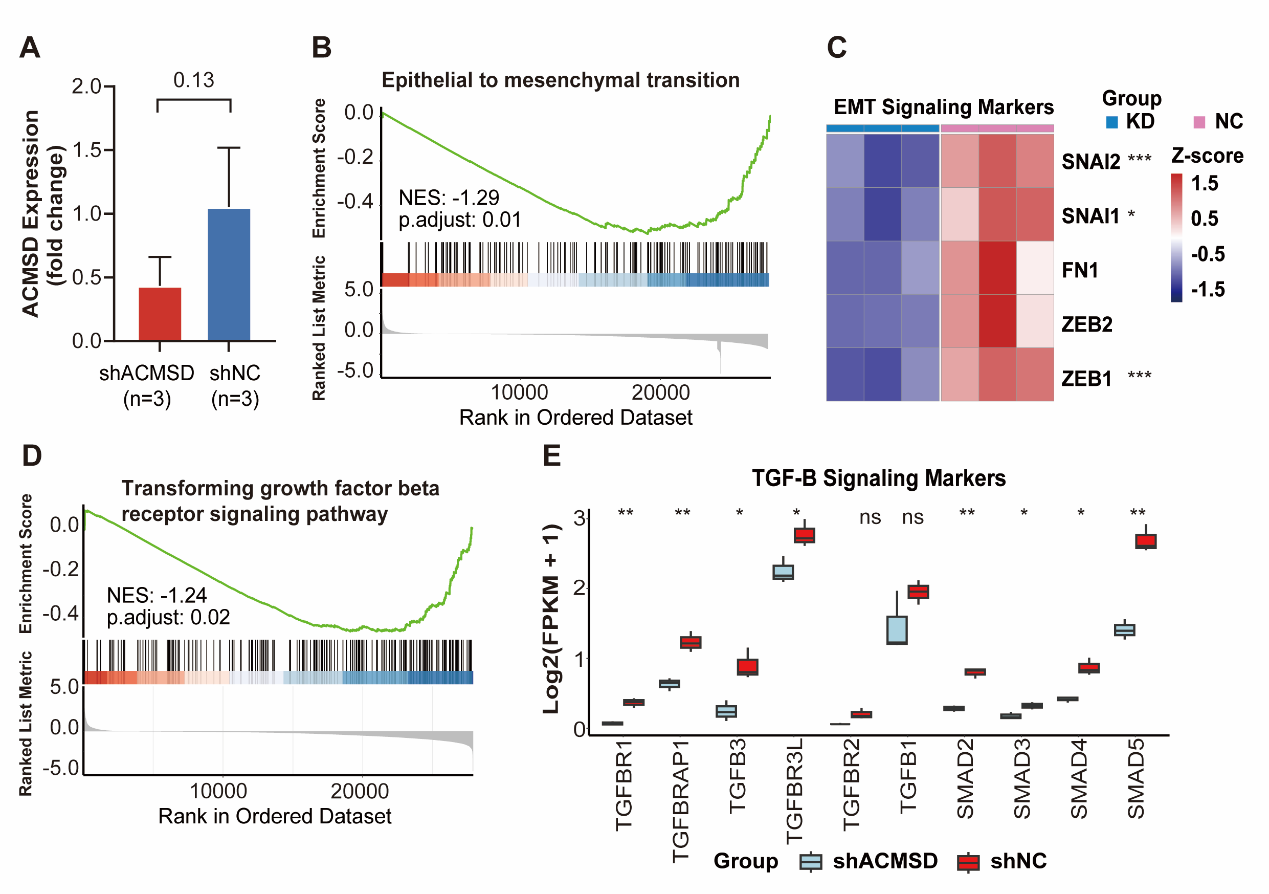


**Figure S6. ACMSD knockdown suppresses the TGF-β/EMT signaling pathway in colorectal cancer cells.**

**(A)** Validation of ACMSD knockdown efficiency. The bar chart shows the relative ACMSD mRNA expression (fold change) in NCI-H716 cells transfected with ACMSD-targeting shRNA (shACMSD) or scramble shRNA (shNC) (n = 3 per group).

**(B)** Gene Set Enrichment Analysis (GSEA) plot demonstrating the significant suppression of the epithelial-to-mesenchymal transition (EMT) pathway based on RNA-Seq data from shACMSD and shNC cells.

**(C)** Heatmap of signature markers associated with EMT. The heatmap displays Z-score normalized expression levels of key EMT-related genes. Red indicates higher expression, and blue indicates lower expression.

**(D)** GSEA plot illustrating the downregulation of the Transforming growth factor beta (TGF-β) receptor signaling pathway following ACMSD knockdown.

**(E)** Expression levels (Log_2_(FPKM+1) of key TGF-β signaling markers in shACMSD and shNC cells (n = 3 per group). Box plots show the distribution of gene expression. Statistical significance was determined by t-test. **P* < 0.05; ***P* < 0.01; ****P* < 0.001; *****P* < 0.0001; ns, not significant.


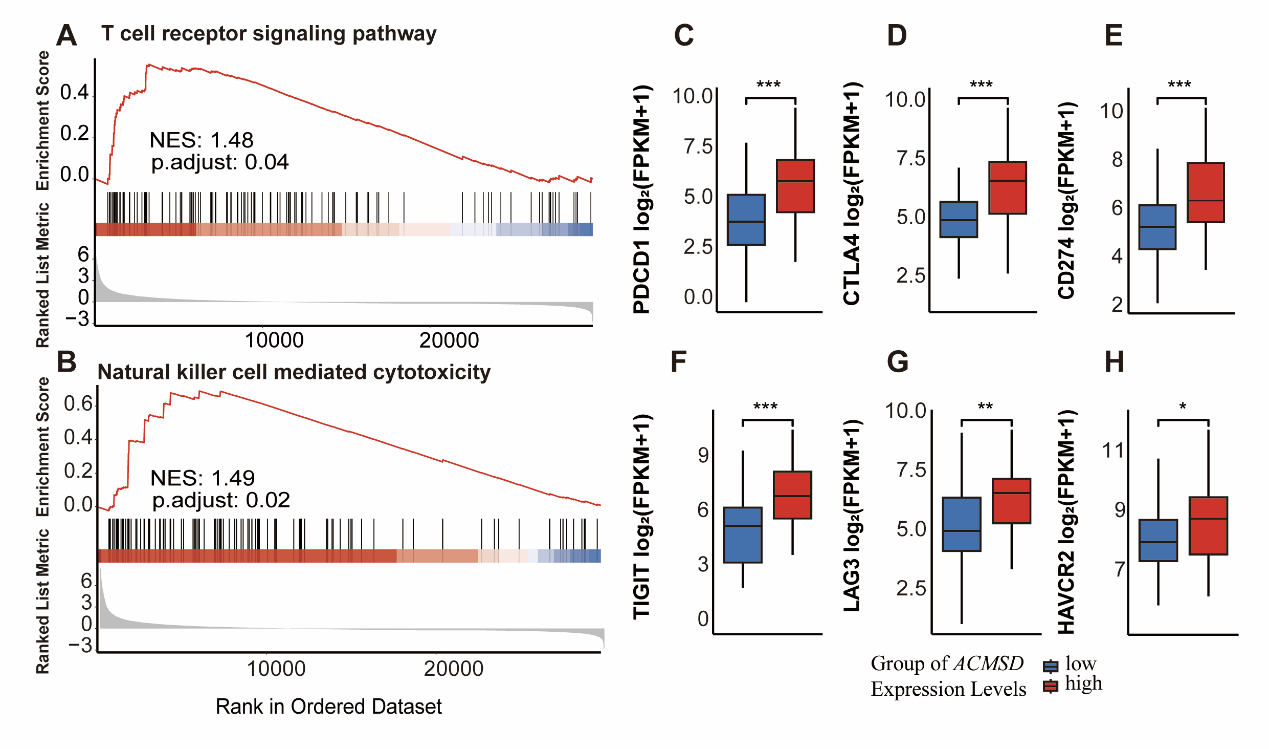


**Figure S7. Positive correlation of ACMSD expression with immune effector pathways and immune checkpoint molecules in CRLM.**

(A, B) GSEA enrichment plots of the T cell receptor signaling pathway (A) and the natural killer cell-mediated cytotoxicity pathway (B) in CRLM samples with high ACMSD expression compared to those with low ACMSD expression.

(C–H) Boxplots showing the expression levels of selected immune checkpoint inhibitor (ICI) genes between high and low ACMSD expression groups in CRLM samples. PDCD1 (C, encoding PD-1**), CTLA4 (D), CD274 (E, encoding PD-L1), TIGIT (F), LAG3 (G), and HAVCR2 (H, encoding TIM-3). Statistical significance between groups was determined by t‑test. **P* < 0.05; ***P* < 0.01; ****P* < 0.001; *****P* < 0.0001; ns, not significant.


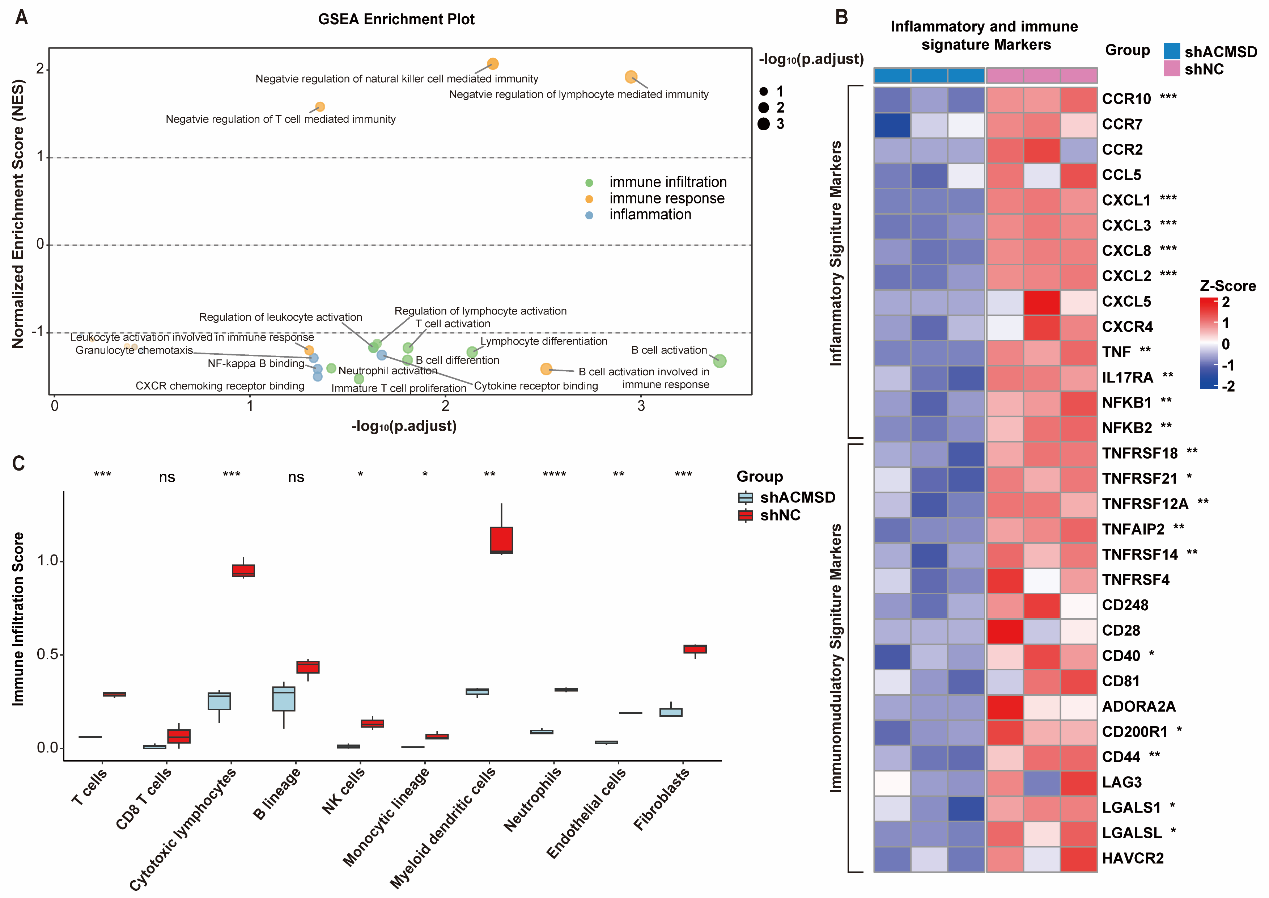


**Figure S8. ACMSD knockdown suppresses tumor-intrinsic immunomodulatory programs and inflammatory signaling.**

1. Gene Set Enrichment Analysis (GSEA) of shACMSD and shNC cells. The dot plot displays the Normalized Enrichment Score (NES) and statistical significance (-log10 (p.adjust)) of various immune-related pathways. Pathways associated with immune infiltration, immune response, and inflammation are highlighted with distinct colors.
2. Heatmap depicting the comparative expression of tumor-derived inflammatory and immunomodulatory signature markers between shACMSD and shNC cells. The heatmap shows the Z-score normalized expression levels. Red indicates higher expression, and blue indicates lower expression.
3. Box plot illustrating the activation levels of immune-related gene expression programs, as estimated by the MCP-counter algorithm, in shACMSD and shNC cells. These scores reflect tumor-intrinsic transcriptomic programs associated with immune modulation rather than actual immune cell counts.

Statistical significance between groups was determined by t‑test. **P* < 0.05; ***P* < 0.01; ****P* < 0.001; *****P* < 0.0001; ns, not significant.
