# Supplementary figures and images for: DeepMetabio-mCRC Screener: A Multi-Omics Deep Learning Framework for Early Risk Prediction and Biomarker Discovery in Colorectal Liver Metastasis
Source: Comput Struct Biotechnol J. 2026 May 25;35(1):0074. doi: 10.34133/csbj.0074 (PMC13199651; doi:10.34133/csbj.0074)

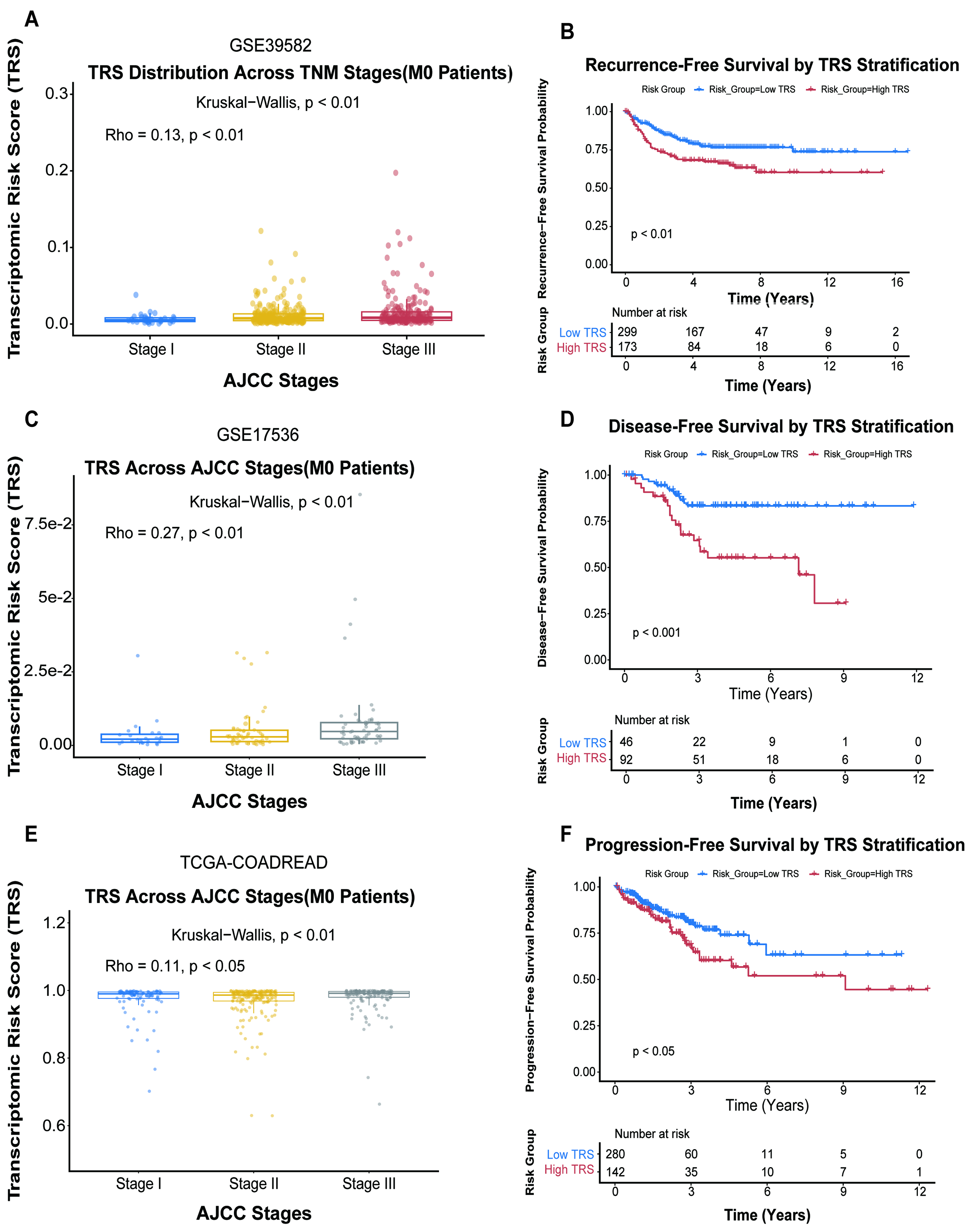

Supplement: Supplementary 1 — Figs. S1 to S8 Tables S1 to S27 [file csbj.0074.f1.zip › FIgure S3.tif]

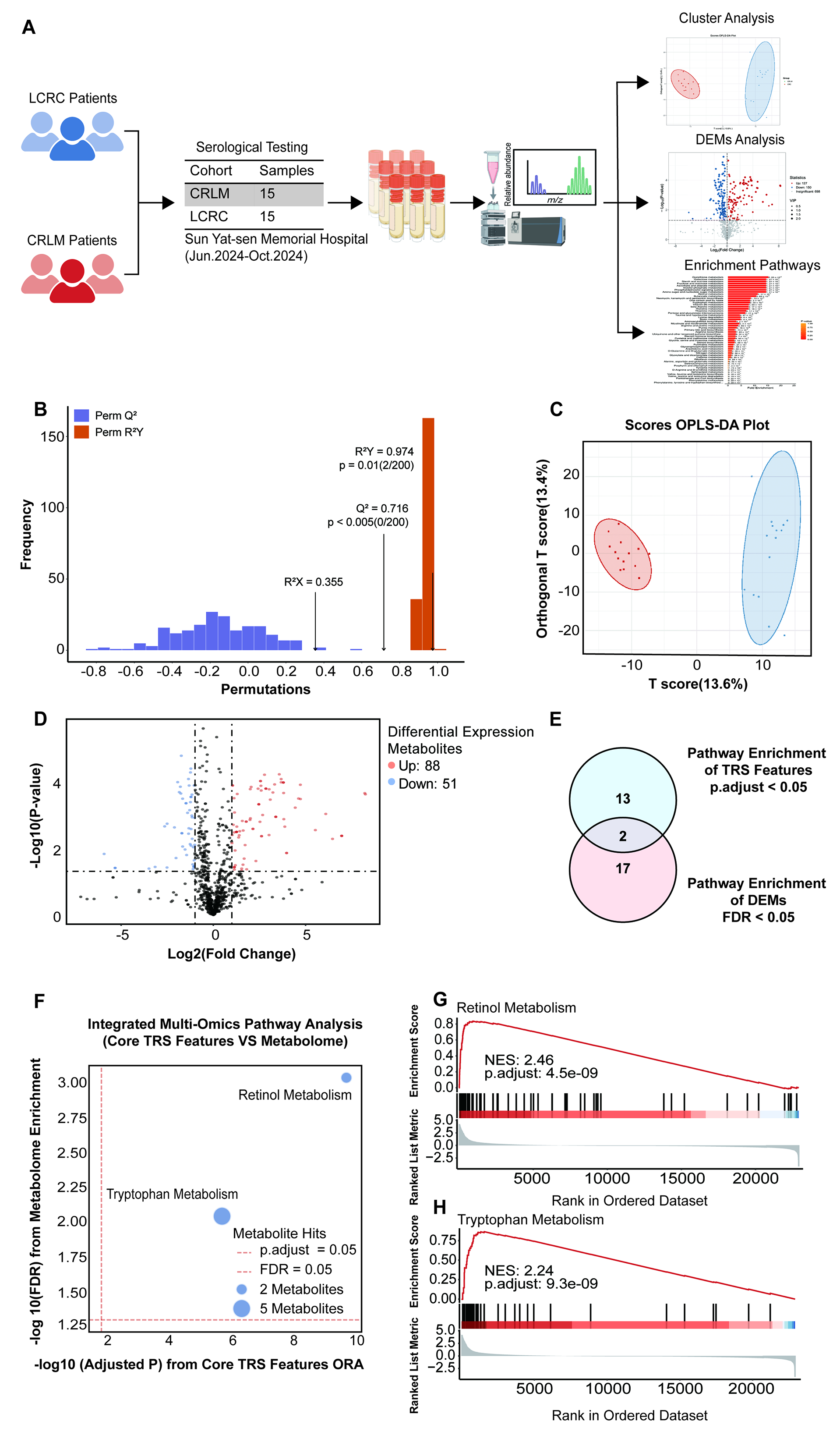

Supplement: Supplementary 1 — Figs. S1 to S8 Tables S1 to S27 [file csbj.0074.f1.zip › Figure S4.tif]

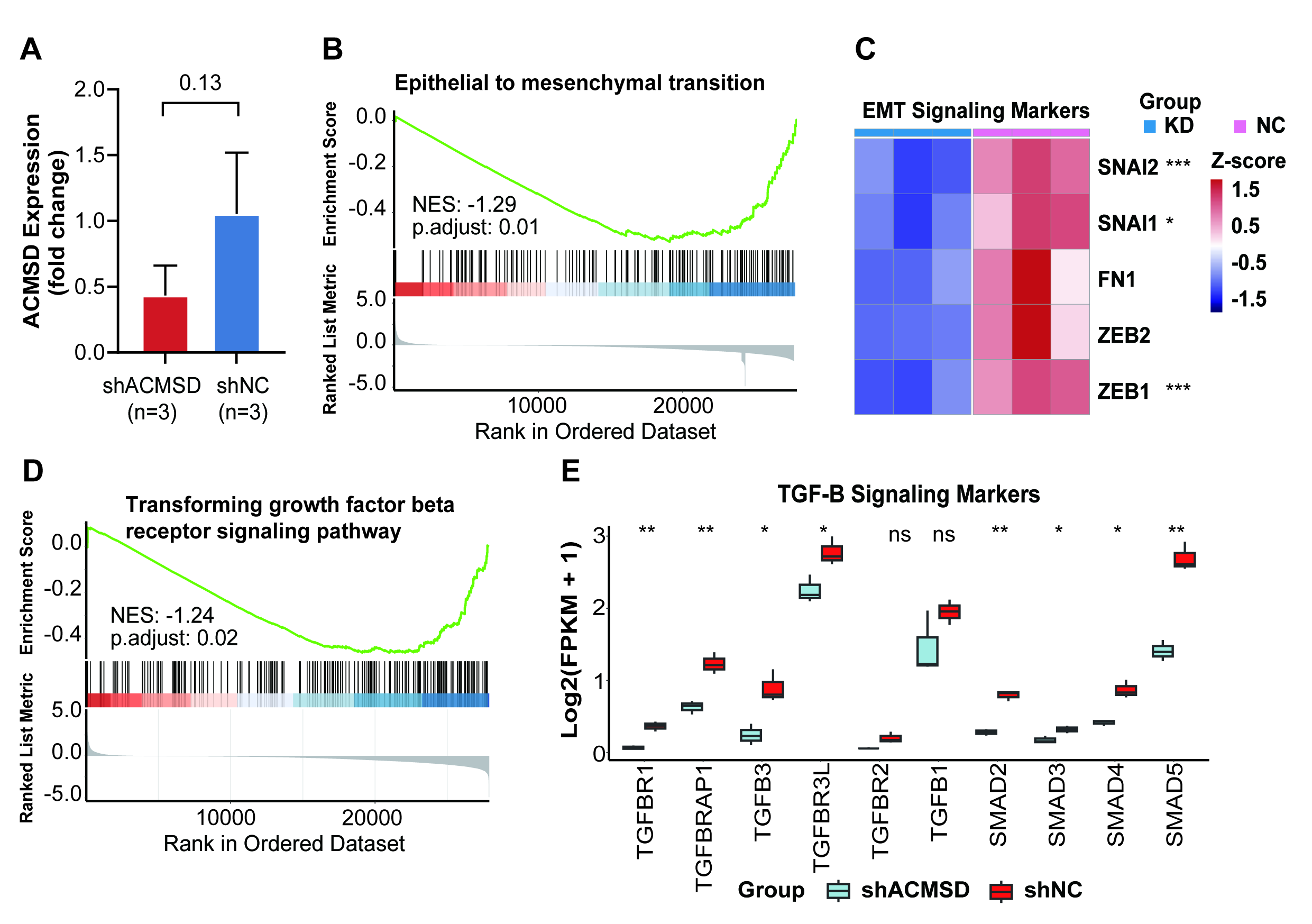

Supplement: Supplementary 1 — Figs. S1 to S8 Tables S1 to S27 [file csbj.0074.f1.zip › Figure S6.tif]

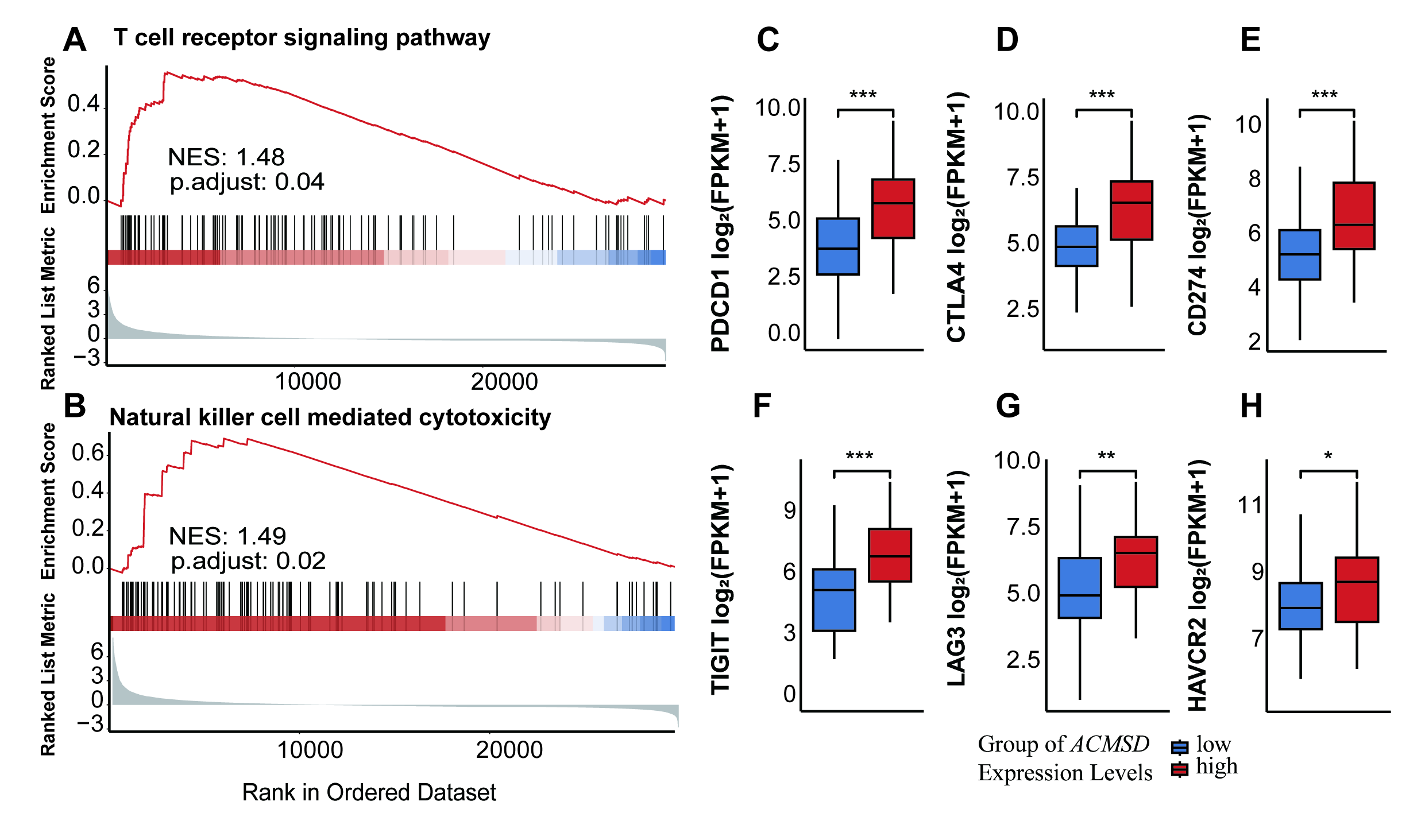

Supplement: Supplementary 1 — Figs. S1 to S8 Tables S1 to S27 [file csbj.0074.f1.zip › Figure S7.tif]

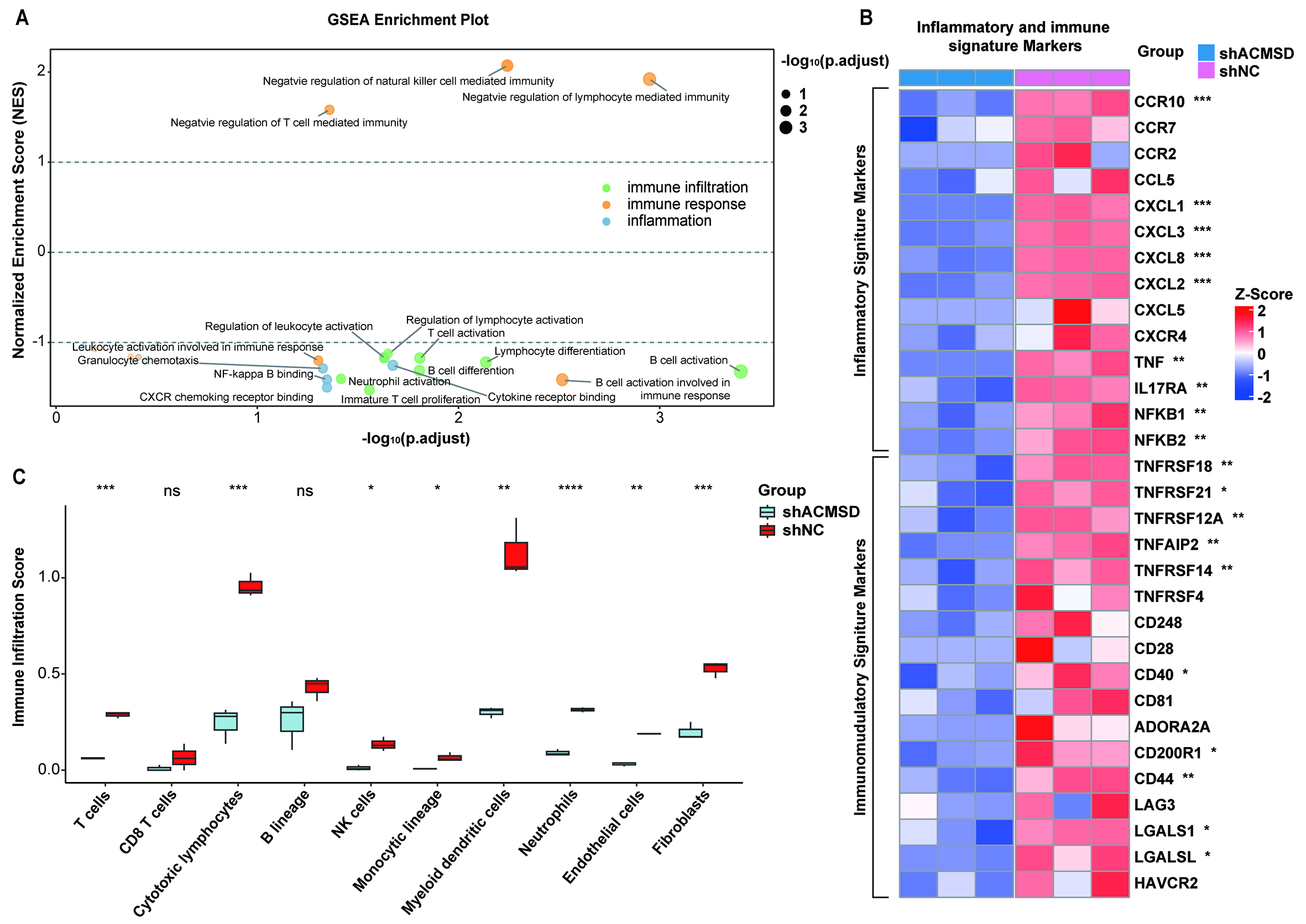

Supplement: Supplementary 1 — Figs. S1 to S8 Tables S1 to S27 [file csbj.0074.f1.zip › Figure S8.tif]

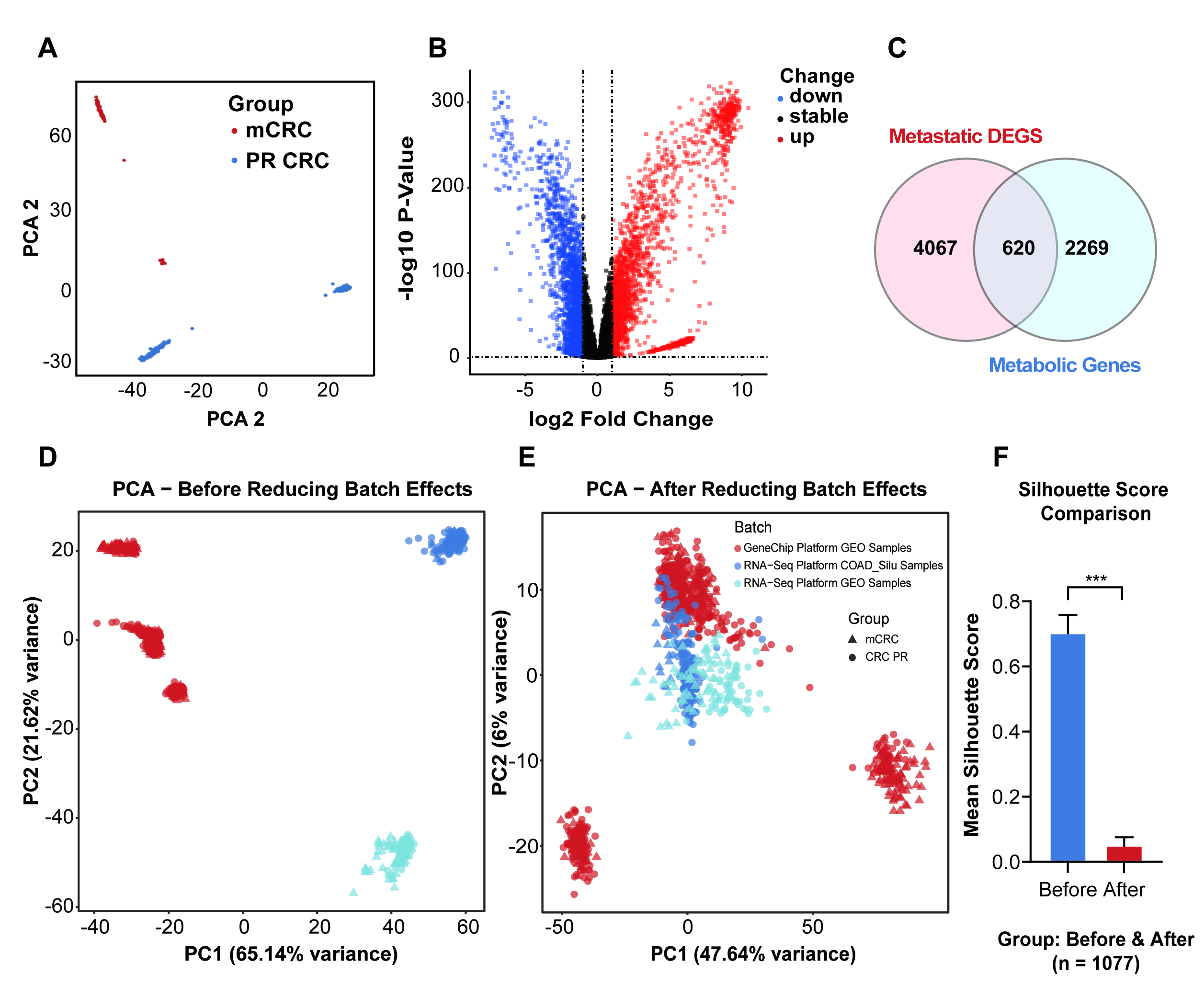

Supplement: Supplementary 1 — Figs. S1 to S8 Tables S1 to S27 [file csbj.0074.f1.zip › Supplementary Figure 1.tif]

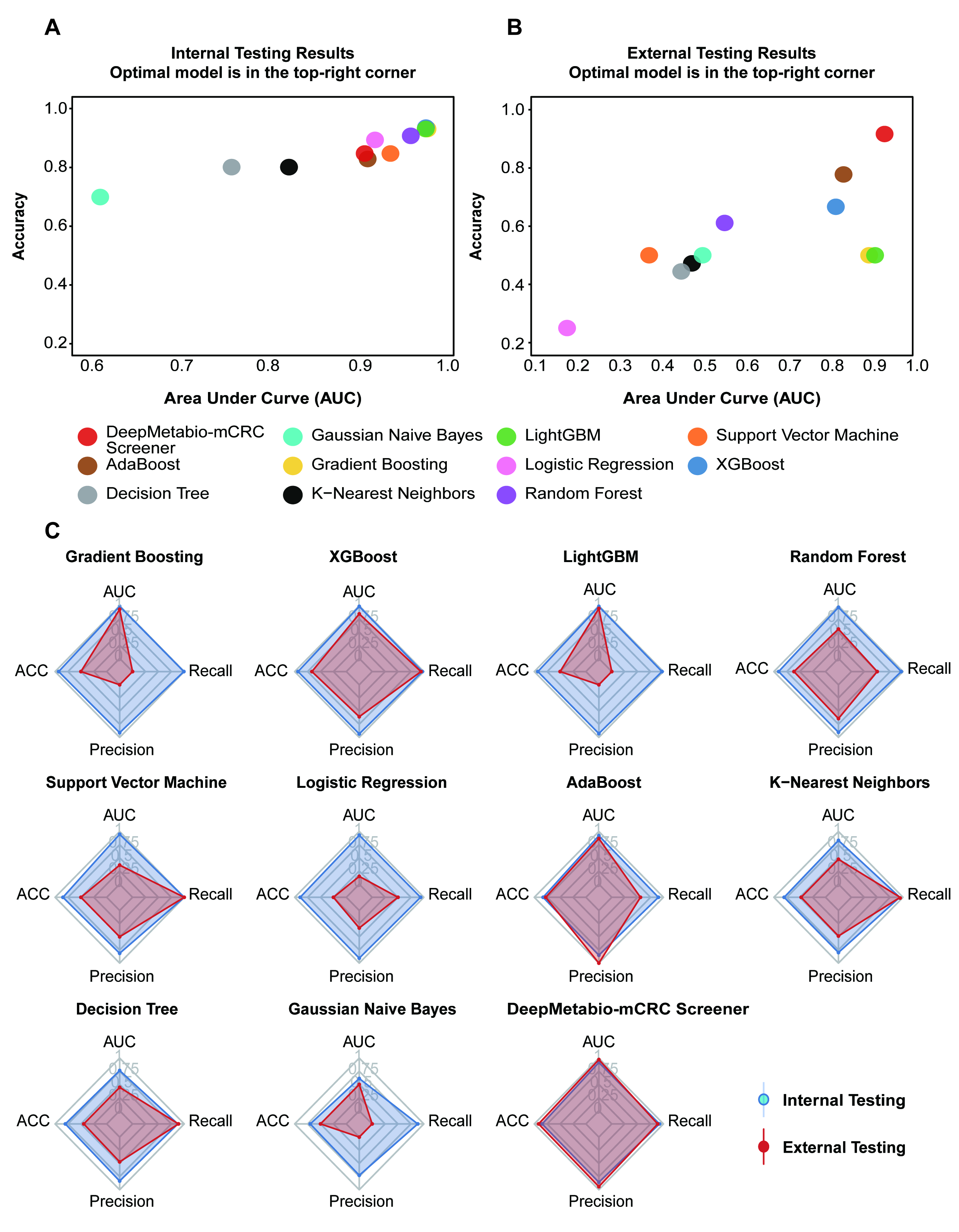

Supplement: Supplementary 1 — Figs. S1 to S8 Tables S1 to S27 [file csbj.0074.f1.zip › Supplementary Figure 2.tif]
